# Urban effects on local cloud patterns

**DOI:** 10.1073/pnas.2216765120

**Published:** 2023-05-15

**Authors:** Thuy Trang Vo, Leiqiu Hu, Lulin Xue, Qi Li, Sisi Chen

**Affiliations:** ^a^Department of Atmospheric and Earth Science, The University of Alabama in Huntsville, Huntsville, AL 35805; ^b^Research Applications Laboratory, National Center for Atmospheric Research, Boulder, CO 80301; ^c^School of Civil and Environmental Engineering, Cornell University, Ithaca, NY 14853

**Keywords:** cities, cloud climatology, remote sensing, CONUS

## Abstract

Urbanization extensively modifies surface roughness and properties, impacting regional climate and hydrological cycles. Urban effects on temperature and precipitation have drawn considerable attention. These associated physical processes are also closely linked to clouds’ formation and dynamics. Cloud is one of the critical components in regulating urban hydrometeorological cycles but remains less understood in urban-atmospheric systems. We analyzed satellite-derived cloud patterns spanning two decades over 447 US cities and quantified the urban-influenced cloud patterns diurnally and seasonally. The systematic assessment suggests that most cities experience enhanced daytime cloud cover in both summer and winter; nocturnal cloud enhancement prevails in summer by 5.8%, while there is modest cloud suppression in winter nights. Statistically linking the cloud patterns with city properties, geographic locations, and climate backgrounds, we found that larger city size and stronger surface heating are primarily responsible for summer local cloud enhancement diurnally. Moisture and energy background control the urban cloud cover anomalies seasonally. Under strong mesoscale circulations induced by terrains and land–water contrasts, urban clouds exhibit considerable nighttime enhancement during warm seasons, which is relevant to strong urban surface heating interacting with these circulations, but other local and climate impacts remain complicated and inconclusive. Our research unveils extensive urban influences on local cloud patterns, but the effects are diverse depending on time, location, and city properties. The comprehensive observational study on urban–cloud interactions calls for more in-depth research on urban cloud life cycles and their radiative and hydrologic implications under the urban warming context.

## Significance

Clouds play a key role in radiation and precipitation processes in the climate system. Urban-modified surface properties as well as anthropogenic heat and aerosols can alter cloud processes. How and to what extent cities interact with background conditions to impact local-regional cloud patterns remain poorly understood. Using long-term and large-scale satellite cloud observations over 447 US cities, this observational-based urban–cloud interaction research reveals cloud enhancements for most cities during warm seasons, even for smaller cities. We also found ubiquitous urban effects on cloud patterns climatologically primarily determined by climate backgrounds interacting with cities and urban surface heating. These findings advance our understanding of urban effects on regional atmospheric systems and highlight further research needs in urban hydrometeorological processes.

Clouds modulate the atmosphere–surface radiation balances and possibly form precipitations, which play key roles in Earth’s energy and water cycles (1). Clouds are complex and highly variable in the atmosphere (1, 2). Worldwide urbanization considerably modifies the local surface properties and roughness and emits heat and anthropogenic aerosols into the atmosphere. These modifications lead to rather complex changes in urban land–atmosphere interactions, resulting in unique urban climate phenomena (3–6). Previous studies have extensively shown that cities alter local temperature (5, 7) and precipitation (e.g., ref. 8). The urban effect on changing cloud frequencies was also reported over a handful of large cities (9–12). In such complex systems, the potential impacts on urban cloud patterns are not a surprise, but whether, how, and to what extent cities alter the local clouds still remain poorly understood. Changes in local cloud patterns, directly and indirectly, affect the energy (through radiation) and water (through precipitation) cycles in the urban system, which have important implications for urban heat, air quality, and flash floods that matter to urban populations.

**Fig. 1.**
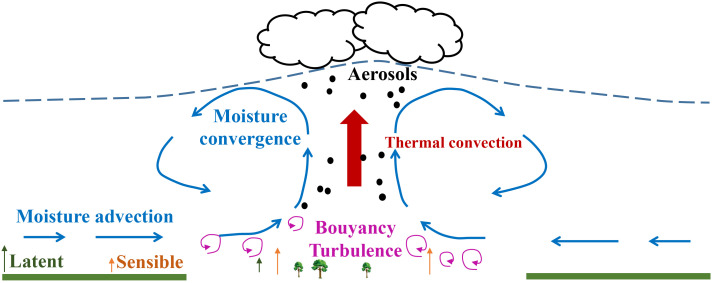
Possible pathways contributing to urban cloud formation. Urban–rural circulations advect moist air from the surrounding rural areas into the city and form a moisture convergence over the city. Thermal convection induced by surface heating combined with moisture convergence collectively transports moisture to a higher level in the atmosphere and enhances cloud occurrences over the city. Wind field changes due to urban surface roughness interacting with local urban buoyancy influence the convergence. Urban aerosol emission modifies cloud formation and properties.

Conceptually, several possible pathways can contribute to cloud modification over cities (Fig. 1). Imperviousness-dominant urban areas with anthropogenic heat emissions typically have stronger sensible heat partitioning compared to the vegetative rural surroundings (6). The considerable surface heating forms strong convective uplift of available moist air to a higher level, likely developing a higher and more unstable boundary layer during daytime or warm seasons. The increased vertical uplift and moisture convergence collectively enhance the chance of cloud occurrences over cities (9, 11). For example, a recent study analyzed daytime optical satellite observations and in situ ceilometer and confirmed the increased daytime cloud base height and associated enhanced afternoon cloud cover over London and Paris (9); similar phenomena were also observed in Nashville (11). Positive sensible heat fluxes after sunset over cities via heat released from human-made urban fabrics can sustain the vertical mixing and impact cloud formation at night (9). Moreover, a strong urban–rural contrast in surface energy balance can stimulate the urban–rural circulations that transport moisture from wetter rural surfaces to a drier urban atmosphere to sustain the cloud locally under calm conditions (11, 13–15). On windy days, the rougher urban areas increase the surface drag and wake turbulence thereby reducing wind speed at the urban center (3, 16, 17). The wind field changed by the urban roughness interacting with local urban forcing (e.g., buoyancy fluxes) subsequently alters the convergence that can further impact the motion and occurrences of clouds. In addition, abundant cloud condensation nuclei (CCN) from urban aerosol emissions can promote cloud formation, analogous to forest-induced cloud enhancements from biogenic volatile organic compounds (BVOCs) (18). However, aerosols may reduce the cloud lifetime for these highly loaded with small droplets by enhancing the entrainment–evaporation process (19, 20). Thus, aerosol effects on cloud formation remain inconclusive.

Overall, cities’ roles in modifying cloud patterns are complex and can vary spatially and temporally. Cities of different forms and functions (e.g., city size and heat emissions) interacting with the regional background from distinctive geographical locations (e.g., inland/coastal and/or flat/mountainous location) to regional climate (e.g., energy and moisture availability) collectively determine primary physical processes (21), thereby reshaping the local cloud patterns. For example, regionally, moisture availability from different climates determines whether there is a local influence on cloud formation. Also, interactions of urban-induced local changes with persistent meso- to regional-scale circulations like mountain–valley and sea/lake–land breezes can further complicate the processes. So far, a few observational studies suggest that surface heating contributes to the enhancement of urban cloud cover during the summer (e.g., ref. 9). However, there is a lack of systematic understanding of such phenomena and let alone determining the relative roles of local and regional factors (such as urban form, regional climate, and geographic locations) acting simultaneously on changing local clouds. Here, we analyzed the diurnal and seasonal cloud patterns over 447 cities across the Contiguous United States (CONUS) using nearly two-decade (2002 to 2020) high-resolution (1-km-pixel) subdaily cloud product from the Moderate Resolution Imaging Spectroradiometer (MODIS). By statistically linking the urban cloud cover and frequency anomalies with local surface properties and climate features, this study aims to answer the following key research questions: 1) How do cities modify local cloud patterns? And 2) how do regional climate and local urban properties influence the diurnal and seasonal changes of urban cloud patterns?

## Urban-Modified Cloud Pattern

We estimated the spatial differences of cloud cover over 447 pairs of cities and their adjacent environmental background across CONUS at the monthly scale (*Δ*_*CloudCover*_) and counted the frequency ( *f*_+_) of cloud-enhanced days (+*Δ*_*CloudCover*_) relative to the total days (*Materials and Methods*). The studied cities represent a wide range of geographic backgrounds and are grouped into three categories: inland (217), coastal (127), and mountainous (103) cities (*SI Appendix*, Fig. S1*A* for the distribution of cities in three categories), which are located within three major climate regions defined by the Köppen-Geiger climate classification (22) (namely, arid, cold, and temperate climates).

**Fig. 2.**
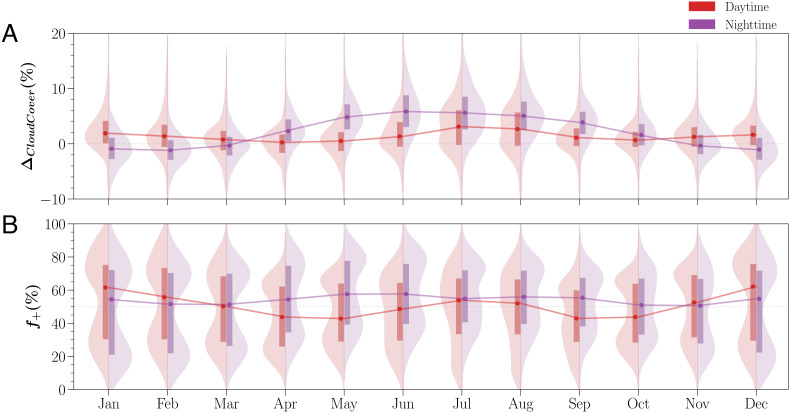
Temporal dynamics of urban modified cloud patterns across CONUS cities. Eighteen-year monthly (*A*) *Δ*_*CloudCover*_ and (*B*) cloud enhancement frequency *f*_+_ of studied CONUS cities are summarized in side-by-side density curves and boxes for day (*Left*) and night (*Right*), respectively. The solid lines connect the monthly median values of all city signals.

Urban signals of cloud cover and occurrence are extensively observed among cities of all sizes but exhibit a remarkable diurnal and seasonal variation (Fig. 2). A majority of cities have significant *Δ*_*CloudCover*_ (80% for day and 90% for night, in July) (also in *SI Appendix*, Fig. S4). Most of those significant cities experience more cloudiness for both days (e.g., 78% cities with positive signals in July) and nights (96%) during warm months (i.e., May–September), reaching the monthly maximal spatial enhancements in July day at about 3.1% (the median *Δ*_*CloudCover*_ of all studied cities) and June night at 5.8%.

**Fig. 3.**
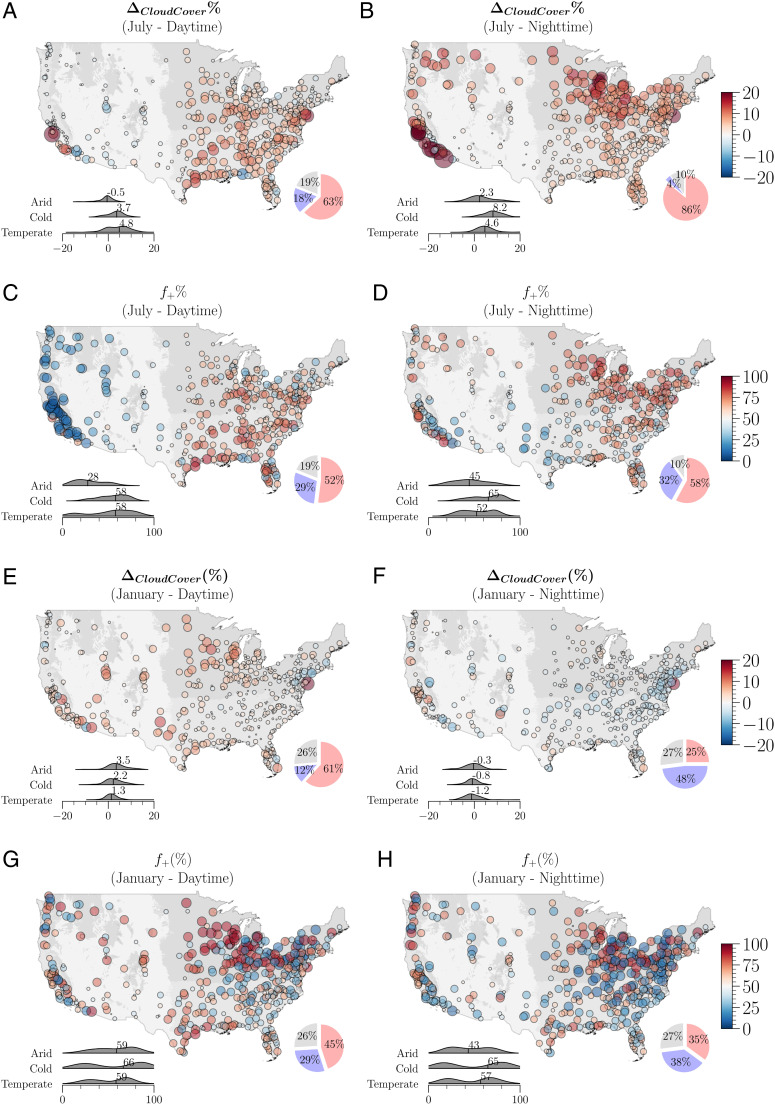
Spatial variations of urban-modified cloud cover (*Δ*_*CloudCover*_) and frequencies (*f*_+_) across CONUS diurnally and seasonally, for July (*A*–*D*) and January (*E*–*H*). Each circle represents a city. Its color indicates the intensity of the estimated variable, and its size is proportional to |*Δ*_*CloudCover*_| or |*f*_+_ − 50%|. A distribution comparison among three climate regions (arid, cold, and temperate) is shown in the *Lower* *Left* corner as density curves, and the median value for each region is labeled. The ratios of studied cities with +*Δ*_*CloudCover*_ (*f*_+_ > =50%) are shown in red and −*Δ*_*CloudCover*_ (*f*_+_ < 50%) in blue at the lower right corner for cities with significant *Δ*_*CloudCover*_ at *P* = 0.05 level. The ratios of studied cities with insignificant results are shown in gray.

Despite a relatively small magnitude of spatial cloud enhancement, the actual spatial difference in given days can be still considerable. Partly, 18-y monthly average accounts for all scenarios, including partial cloudy (±*Δ*_*CloudCover*_), overcast (*Δ*_*CloudCover*_ = 0), and clear skies (*Δ*_*CloudCover*_ = 0), which dampens the long-term averaged spatial differences (*SI Appendix*, Fig. S2 for the probability density function of daily *Δ*_*CloudCover*_). In addition, urban enhanced cloudiness (+*Δ*_*CloudCover*_) occurs about or slightly less than 50% (*f*_+_) of days in warm months. The instantaneous cloud spatial enhancements are expected to be stronger to pick up the positive signal in the long-term average (Fig. 2; also, see the western CONUS in Fig. 3 *A* and *C*).

In general, enhanced cloud cover at night almost doubles the daytime effect during warm months (Fig. 2*A*). During the warm season, despite the drier urban areas during the day (23, 24), the overall stronger radiation encourages regional moisture through evapotranspiration into the atmosphere as well as moisture transport through large-scale circulations e.g., low-level jet (25). Locally, urban–rural thermodynamic contrasts and surface roughness during summers provide persistent turbulent updrafts and stronger mixing in the boundary layer. These generate stronger vertical motion lifting the available moisture over cities to the cloud condensation layer (9, 11). Comparably, cities have stronger urban surface heating at night while being likely moister than the regional climate background (24, 26, 27). The continuous vertical mixing and relatively higher moisture level provide more favorable conditions for cloud formation over cities at night.

The spatial distributions of daytime/nighttime urban cloud enhancement during summers, nevertheless, are rather different (Fig. 3 *A* and *B*). For instance, daytime urban cloud enhancements in both spatial cover and frequency of enhancement are stronger over eastern and southeastern CONUS. Nocturnal enhancement signals are more manifest over the cold climate region at the northern CONUS as well as the west coast where major metropolitan regions are located. Overall, cities with moist warm-season climates tend to show stronger local cloud enhancement as compared to arid regions. These diurnal contrasts and spatial clusters indicate the potential influences of the interactions of cities and regional climates on the cloud processes.

The winter signal of *Δ*_*CloudCover*_ has a weaker but distinct diurnal pattern compared to summertime. Daytime urban clouds are spatially enhanced by 1.8% in January (Figs. 2*A* and 3 *A* and *E*), compared to 3.1% in July (Fig. 2*A*). Interestingly, summer strong daytime enhancement in southern regions becomes much weaker during winter, and arid regions show the strongest local cloud cover enhancement. Less cities with significant *Δ*_*CloudCover*_ in winter months (*SI Appendix*, Fig. S4, i.e., 74% for day and 73% for night in January) as compared to warm months. Within these cities, 84 % of these cities have weak daytime cloud enhancements (Fig. 3*E*), and in contrast, about 66% of cities are slightly more clear on winter nights (−0.9% to −1.2% from December to February) (Fig. 3*F*). Winter months featured lower surface temperature, and a stabler boundary layer, particularly at night, may form favorable conditions for fogs or low stratus when there are sufficient moisture and aerosols. The relatively higher temperature in cities, particularly at night, may suppress fog formation, resulting in fog holes in cities, which have been observed in the Indo-Gangetic Plains (28). In addition, we found obvious regional clustering of cloud cover changes (i.e., normal distribution), but *f*_+_ during the cold seasons are more complex spatially (Fig. 3 *G* and *H*), and studied cities also exhibit a bimodal distribution in the enhanced cloud frequency (Fig. 2*B*). During the transition months (i.e., April and October), the CONUS-wide median *Δ*_*CloudCover*_ are close to zero but with mixed weak positive and negative signals of *Δ*_*CloudCover*_ (Fig. 2*A* and *SI Appendix*, Figs. S5–S8).

## Influences of Local and Regional Factors on Urban Cloud Patterns

**Fig. 4.**
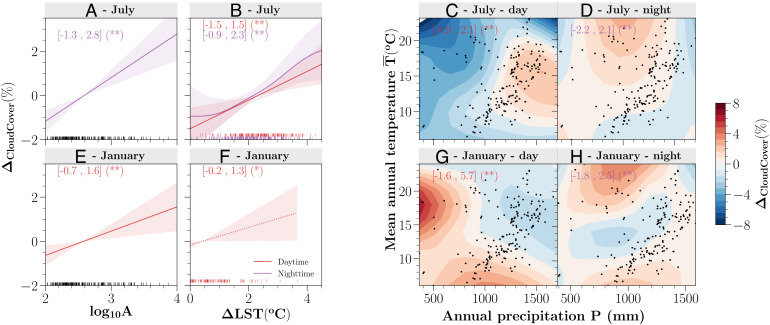
Estimated effects of local and regional properties on local cloud patterns in GAMs for inland cities in July and January. The effects of city size [*log*_10_*A*, in *log*_10_(*km*^2^)] (*A* and *E*), surface heating (*ΔLST*, in ° C) (*B* and *F*), and regional climate (*P*, in mm and $\bar{T}$, in ° C) (*C*, *D*, *G*, and *H*) are shown. The standard errors of combined effects *P* & $\bar{T}$ can be found in *SI Appendix*, Fig. S15. Only statistically significant results are illustrated: dashed lines for *P* = 0.05 level (*) and solid lines for *P* = 0.01 level (**). The shaded regions indicate 2 standard errors predicted by each factor. The minimum and maximum estimated effects and the significant level on *Δ*_*CloudCover*_ (%) of all studied cities are labeled in brackets for day and night, respectively. The vertical bars at the *bottom* in *A*, *B*, *E*, and *F* suggest the sampled cities. Black dots are shown for the locations of cities (*C*, *D*, *G*, and *H*). Note that the estimated results show only +*ΔLST* values (*B* and *F*). The log-scale inland city size ranges from 1.95 (Manhattan, Kansas) to 4.02 (Atlanta, Georgia) in *log*_10_*A* (*A* and *E*). Only cities with significant *Δ*_*CloudCover*_ at *P* = 0.05 level are analyzed.

**Fig. 5.**
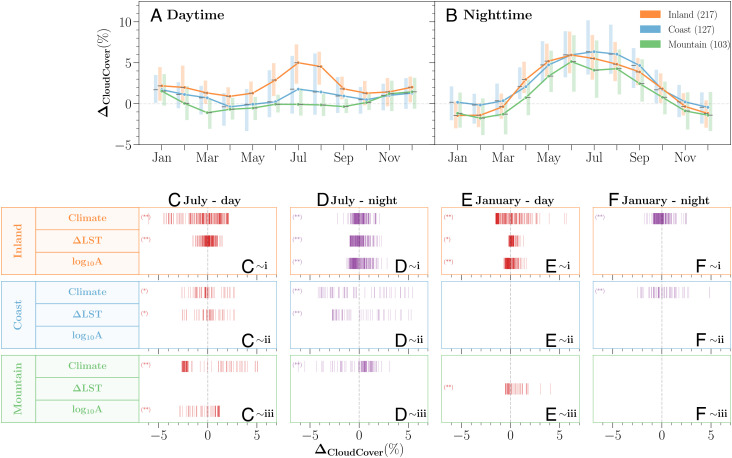
(*A* and *B*) Seasonal and diurnal variations of urban-modified cloud patterns *Δ*_*CloudCover*_ summarized in three geographical regions and (*C*–*F*) summary of estimated effects of local [city size (*log*_10_*A*) and surface heating (*ΔLST*)] and regional (regional climate, *P* and $\bar{T}$) effects on the spatial variations of urban cloud anomalies for inland (*i*), coastal (*ii*), and mountainous (*iii*) cities. Boxes show the median (solid line) and interquartile range of *Δ*_*CloudCover*_. (*C*–*F*) Statistically significant results are illustrated with *P* = 0.05 (*) and *P* = 0.01 (**) levels. Vertical bars represent the estimated effect of the studied cities. Only cities with significant *Δ*_*CloudCover*_ at *P* = 0.05 level are analyzed.

The observed modification of urban cloud patterns suggests possibly collective influences from the regional energy and moisture availability for cloud formation as well as the local impacts, such as urban forms (roughness and surface heterogeneity) and functions (anthropogenic emission) that contribute to thermal, chemical, and dynamic effects to these processes. To quantify the relative roles of these drivers in altering urban cloud patterns, we statistically analyze factors that are associated with cloud processes, namely, city size (log_10_*A*) as a surrogate indicator of surface roughness and anthropogenic emissions, differential surface heating measured by land surface temperature (LST) (*ΔLST*), moisture availability (annual precipitation, *P*), and energy availability (mean annual temperature, $\bar{T}$), in a series of generalized additive models (GAMs) (*Materials and Methods*, section *Statistical Modeling*). The effects of city size and urban surface heating are often assumed linearly related (29, 30), but this relationship is not necessarily applicable to cloud formation. For instance, larger cities are often aerodynamically rougher and have a greater amount and variety of anthropogenic emissions as compared to smaller cities, while differential surface heating that is associated with the moisture advection and convergence has been found primarily controlled by the regional climate background (29). Thus, we consider the GAMs to capture the possible nonlinear effects. Further, to isolate the persistent influences from the mesoscale circulations induced by orography and land–water contrasts, we separate the analyses for cities at inland, coast, and mountains. The summary of GAM results on diurnal and seasonal contributions of influential factors to urban cloud enhancement or reductions is shown in Fig. 4 for inland cities and in Fig. 5 for three geographic groups in January and July (*SI Appendix*, Figs. S9–S24).

### Inland Cities without Terrain Influences.

Although both summer daytime and nighttime cloud enhancement are observed for most inland cities (Fig. 4*A* –*D* for July, *SI Appendix*, Figs. S10–S12 for their spatial distributions, and Fig. 5 *A* and *B* for their spatial variations), their major contributing factors are not the same. During the daytime, wet regions tend to have more urban cloud enhancements while that effect becomes most manifest in the moderate temperature (i.e., energy) region (up to 2.1% local cloud enhancement). The cooler or hotter regions regardless of the moisture availability during the summer day reduce the urban cloud cover (*SI Appendix*, Fig. S14*B* for the separate effect of temperature). The local urban properties show a moderate impact on cloud enhancement linearly. For example, stronger urban surface heating is associated with up to 1.5% daytime cloud enhancement. However, these urban properties become the most relevant contributors to the prevalent nocturnal cloud enhancement (up to 2.3% from surface heating; or up to 2.8% from urban size) (Fig. 4 *A* and *B* for July and *SI Appendix*, Figs. S10 and S11 for other summer months). The regional temperature or precipitation alone, on the other hand, shows no statistical significance at night (*SI Appendix*, Figs. S13 and S14), but a moderately moist and hotter nocturnal climate interacting with cities still promotes more local cloud cover up to 2.1% (Fig. 4*D*).

The divergent diurnal urban effects on cloud cover in wintertime have been found to be more related to the regional temperature pattern and its interaction with the moisture conditions (Fig. 4 *G* and *H* for January, *SI Appendix*, Fig. S12 for other winter months, and Fig. 5 *A* and *B* for their spatial variations). For example, cities interacting with regional climate can enhance local cloud cover by up to 5.7% during the day and reduce up to 1.8% at night. Cooler and moist regions interacting with cities tend to produce more urban clouds during winter daytime. Temperature exerts a stronger impact relative to the regional moisture (*SI Appendix*, Figs. S13 and S14*A*), and colder northern regions show more urban cloud cover. Similar to the summer effect, we have found here the enhanced urban surface heating and urban size positively contribute to the enhanced local clouds, but at relatively weaker magnitudes (Fig. 4 *E* and *F* for January, and *SI Appendix*, Figs. S10 and S11 for other winter months) (up to 1.6% and 1.3% from urban size and surface heating, respectively). In the arid region, a few sampled cities indicate that mild winter balanced with moderate moisture also yields more cloud contrast (Fig. 4*G*). Reduction in winter nighttime clouds has been observed to be more related to the regional climate, rather than the impact of local urban properties (i.e., urban size and surface heating results are not statistically significant). The temperature effect and its interactions with the moisture sources and cities show more complicated patterns.

### Coastal and Mountainous Cities Under Persistent Mesoscale Influences.

The urban effects on cloud patterns are further modified under mesoscale influences. Unlike similar shared roles of each contributing factor on localized clouds over inland cities, the effect of enhanced surface heating tends to interact more with regional sea–land/mountain–valley breezes, which is responsible for the weakened daytime but still considerable nighttime urban cloud signals (Fig. 5 *C* and *D*, *ii* for July and *E* and *F*, *ii* for January, *SI Appendix*, Figs. S18–S20 for their spatial distributions and Fig. 5 *A* and *B* for their spatial variations). The regional climate and urban size also contribute to these modified signals, yet, their seasonal and diurnal trends are inconclusive.

First, we observe that a much larger variation of summer urban cloud cover changes among coastal cities (*SI Appendix*, Fig. S17) with weakened daytime urban effects but strengthened nighttime effects. Winter patterns are similar to inland cities but with no clear urban effects at night observed in inland and mountainous cities (*Δ*_*CloudCover*_∼0). Comparably, city size and climate background become less important in modulating the daytime coastal urban cloud patterns (all season). Instead, sea breezes bring moisture over land and interact with rough urban surfaces (relatively to coastal landscape) and enhanced urban surface heating (*SI Appendix*, Figs. S11 and S19), which together contribute to stronger moisture convergence over coastal cities for possible cloud formation (31, 32). The presence of cities weakens the land breezes (32) as a result of strong nocturnal heat storage release in cities that reduces the land–water temperature differences. Under this condition, the strong nocturnal cloud enhancement of coastal cities statistically suggests a considerable contribution from urban surface heating (up to 5.3% in the summer). Drier and cooler climate also encourages more nighttime cloud cover by up to 8.1%. However, regional climate effect is mild in cold seasons. Note that cities are located in more complicated terrains; for example, Pacific coastal metropolises are observed with enhanced cloud cover. Weakened land breezes strengthening urban convection combined with down-slope mountain breezes can intensify the nocturnal cloud development, but these clouds can be at a low level, such as frequent summertime fog.

Mountainous cities exhibit little urban cloud signal during the day except for a weak enhancement in the winter. The nocturnal cloud cover enhancements are manifest in the warm season, similar to cities in other geographic backgrounds (Fig. 5 *A* and *B*). The warmer cities in the valley cause diverse diurnal effects on cloud formation. Thus, we see much contrasting signals of summer clouds (daytime cloud cover reduction and nighttime enhancement) (Fig. 5 *C* and *D*, *iii* for July and *E* and *F*, *iii* for January, *SI Appendix*, Fig. S23 for other summer months, and Fig. 5 *A* and *B* for their spatial variations). Daytime up-slope winds as well as urban convection (33) limit the moisture transport for localized clouds. Here, we found that urban surface heating is considerably contributing to the winter daytime cloudiness, outweighing its effects on inland and coastal cities. Weakened winter valley winds around the city (34) combined with enhanced urban surface heating likely trigger cloud development over cities. For mountainous cities, the individual controlling factor has large seasonal and diurnal variations of contributions to the modified cloud patterns (Fig. 5*C*–*F*, *iii* and *SI Appendix*, Figs. S22–S24), and some complex interactions may not be adequately captured by the statistical models.

## Discussion and Conclusions

Through comprehensive analyses of two-decade cloud cover observations from the satellite over CONUS cities of all sizes, we found a rather universal urban effect on local cloud patterns with diverse diurnal and seasonal variations. Most inland cities experience enhanced cloudiness in the summertime diurnally. Asymmetric diurnal patterns have been found in the wintertime, featured by enhanced daytime and reduced nighttime cloud occurrences. Regional variances of urban cloud anomalies statistically suggest a collective contribution of local urban properties as well as regional climates. Moisture and energy availability interacting with urban systems is the major contributing factor to local cloud pattern changes across seasons. Local urban properties are mainly responsible for summer enhanced cloudiness diurnally. For cities that are subject to persistent mountain–valley and land–ocean/water circulations, the daytime cloud patterns vary considerably during the warm season, but the nocturnal strong cloud cover enhancement remains ubiquitous, partly due to the strong urban surface heating and its interactions with local circulations (31, 32). Overall, the local cloud patterns and associated processes are rather complicated and sometimes inconclusive under the complex geographic background in this study.

The diurnal variability of cloud cover has a significant radiative impact on the Earth’s energy budget (35). At the local-regional scales, uneven cloud distributions may further modify the surface energy balance regulated by land surface properties, leading to diurnal contrasting effects on existing land–atmosphere interactions and feedback. As we have found that the daytime cloud enhancements are partly attributable to urban stronger surface heating. More cloudiness likely reduces the net radiation over warmer urban areas and consequently reduces the existing urban–rural surface heating contrast. This process may negatively impact cloud cycles in a short term. On the other hand, as differential surface heating plays a more critical role in nocturnal cloud occurrences, this enhanced cloud pattern (warm season) facilitates the continuous development of nocturnal heat islands by reducing urban radiative cooling. Therefore, in addition to the primary contributions of urban storage heat release and “cavity” effects from urban canyon structure at the surface level on radiation (e.g., ref. 36), the atmospheric cloud impact can be another source that partly explains the typically stronger nocturnal urban heat island. However, the radiation effects of clouds on the surface can vary substantially based on cloud type and height. This study does not distinguish cloud type and properties specifically. Quantifying the urban-modified cloud patterns on urban heat island climatology need further research, particularly in disentangling the effects under the complex interplay of synoptical conditions and climate and geographic backgrounds.

Urban function may also influence the cloud patterns at the weekly cycle, which directly link to anthropogenic emission of heat and pollutants. Here, we found that the weekly cycles of cloud cover observed in some cities (peaked in March nighttime for 0.39%, median value), but their magnitudes and prevalence are not as clear as seasonal and diurnal variability driven by regional climate background and other urban stationary surface features (*SI Appendix*, Figs. S25 and S26 and section 4 for quantitative results of the analysis at weekly scale). Waste heat emitted from frequent traffics or industrial activities during weekdays can raise temperature more than that of the weekends, leading to the weekly cycles of surface heating (37, 38). A small portion (∼4.4%) of cities shows some weekly variability of surface heating in the growing season, but it remains inconclusive on the contribution of weekly cycles of surface heating to the urban cloud patterns (*SI Appendix*, Fig. S26). For example, weekly signal of surface heating is only −0.04 K for these cities with the significant weekly cloud cycle in March. The potential influences of aerosol due to anthropogenic activities on the cloud weekly variability (e.g., refs. 37, 39, and 40) can contribute to the cloud patterns, but this study does not include this analysis, which is worth future investigation.

Clouds play an important role in the urban hydrological cycle as well, given their strong coupling with precipitation. The improved understanding of urban effects on cloud patterns would thus be beneficial for future urban rainfall quantification research, which remains challenging in both observational and modeling frameworks (8). A better understanding of urban effects on these hydrological-related elements will eventually support urban flash flooding prediction and management in a changing climate (41). The intensity and spatial distributions of event-based rainfall studies suggest uneven occurrences at upwind, downwind, and/or right over the city (8). These modifications are often linked to the balance of differential surface heating and synoptical wind conditions (42) and sometimes aerosol effects (43). For instance, under weak wind and strong thermal differences, the setting can enhance the total precipitation over the entire city. On the other hand, under strong wind and weak thermal contrast, the barrier effect contributes to enhanced rainfall downwind and around the city (44). Interestingly, we observed cloud distributions spatially matching urban extents well (*SI Appendix*, Fig. S1*B*). The climatological cloud signals unveiled from satellite observations account for all synoptical scenarios and all cloud types, and there is no clear preference for cloud occurrences relative to the city and their surrounding areas, although the seasonal prevailing wind may exist. In addition, the effects of aerosols and/or wind field changes due to urban surface roughness in a given geographic background can potentially influence these mechanisms. Urban aerosols can modify the microphysical properties of cloud droplets and thus impact cloud lifetime (e.g., ref. 45), and later the collision-coalescence process leading to raindrop formation (e.g., ref. 46). Finally, depending on geographic locations, background aerosol concentrations combined with urban-induced wind field changes can further complicate the roles of aerosols in the cloud-precipitation processes (8, 43).

Clouds in physical models are widely accepted as sources of uncertainties (e.g., ref. 47). Accurate cloud representations are even more constrained at the urban scale due to complicated local processes in an urban environment. Cloud microphysical and macrophysical processes are complex and often considerably simplified and parameterized in the current mechanistic models. A recent study suggested underestimations of urban cloud cover in a high-resolution numerical weather prediction model over London (15). Our findings show ubiquitous urban effects on cloud patterns among cities of different sizes driven by the complicated interactions of local urban properties and their climate and geographical backgrounds. The urban cloud patterns can thus be a valuable aspect added to existing model prediction assessments and offer insights to improve the key processes of radiation and precipitation in the urban climate system, which is paramount in preparing urban development and services in a changing climate. At the same time, many patterns and relationships found in our study have not been investigated to understand the underlying mechanisms. Detailed modeling and observational studies are required to improve our fundamental understanding of urban–cloud–precipitation–climate interactions.

### Study Domains.

We considered all CONUS cities with more than 50-thousand inhabitants based on US Census Topologically Integrated Geographic Encoding and Referencing (TIGER) 2010 Urban Area product, resulting in 481 cities. After merging the contiguous cities, together we identified a total of 447 urban domains (cities defined in this study). We used the static boundary that is close to the midpoint of 2002 to 2020, and the urbanization rate is on average about 1.1% within the 2010 urban boundaries during the entire studied period (*SI Appendix*, Fig. S29). To disentangle the potentially strong influences of mesoscale circulations (e.g., sea–land and mountain–valley breezes) on cloud formations, we classified these chosen cities into three regional categories: inland (217), coast (127), and mountain (103) (*SI Appendix*, Fig. S1*A*). Coastal cities are those located within 70 km from the shore that are potentially subjected to the dominant sea–land breeze influences (48, 49). Similar to the mesoscale flow assumption for the sea–land breeze, orography can exert persistent and strong circulations (48, 50) that interact with urban areas to further modify the local cloud patterns. Cities located on complex terrains are likely subjected to mountain–valley circulations. Thus, we identified these mountainous cities with a greater than 1,000-m elevation difference with the highest point within 70-km buffered surrounding areas. We used Global Multi-resolution Terrain Elevation Data 2010 (GMTED2010) to identify the elevation. Inland cities are the rest of the noncoastal and nonmountainous cities. CONUS spans a vast area covering a variety of regional climate types. Using Köppen-Geiger climate classification at the first level, the climate backgrounds for all studied urban domains are then grouped into three major categories: cold, temperate, and arid climates (22) (Fig. 3).

### Estimation of Urban-Modified Cloud Patterns.

The subdaily cloud mask layer from MODIS cloud masks (MYD35_L2 C6.1) from 2002 to 2020 is used to identify the cloud patterns. Each overpass of the MODIS onboard Aqua often covers two-hour windows around 13:30 (Aqua-Day) and 1:30 (Aqua-Night) local solar time near the equator, offering the day and nighttime results, respectively. The cloud mask product is generated from a series of classic cloud screening algorithms (51) using a synergy of infrared emittance and visible reflectance, which is widely acceptable for developing other MODIS products and applications. The cloud screening accuracy and uncertainties have been extensively assessed (e.g., ref. 52). Potential biases are reported over ice-snow backgrounds and nighttime and mainly over high-latitude polar regions (53, 54). Our focus is on mid-latitude cities, which are not subject to the strong influences of ice-snow backgrounds. Here, we consider the pixel classified as probably cloudy or cloudy for cloud occurrence and cover in this study. Compared to the geostationary visible-band information for daytime-only analyses in the existing literature, using this relatively high-resolution and subdaily cloud cover largely improves the climatological understanding of diurnal and seasonal changes in cloud patterns at the city scale.

The urban-induced cloud pattern changes (*Δ*_*CloudCover*_) were quantified as the spatial differences of cloud coverage in percent between an urban area and its surrounding background. Cloud coverage is estimated as a fraction of cloudy areas identified by the cloud mask layer relative to the corresponding domain area. The daily *Δ*_*CloudCover*_ then were aggregated at a monthly scale over 18 y at each given urban domain. We conducted the t test to identify the significant urban *Δ*_*CloudCover*_ signals for individual cities in each given month over 18 y at *p* = 0.05 level (*SI Appendix*, Figs. S3 and S4 and section S2 for the detail descriptions and quantitative results of the significance test). The urban areas are defined using US Census urban areas product, and the background is each urban domain’s surrounding region but excludes a transition buffered area to avoid urban-influenced clouds in upwind and downwind directions in the atmosphere. The transition and background areas are buffered proportional to the urban size (*SI Appendix*, Fig. S27 for the specific definition and an example of the transition zone and the background reference). Large water bodies (water bodies with an area larger than 1 km^2^) also have strong local effects on clouds (*SI Appendix*, Fig. S28); thus, we excluded waterbodies across the urban-background domains to avoid nonurban influences (55). The nonurban reference can reach up to five times the urban area. There are about 6% of total studied cities that have a similar size or smaller reference domain due to proximity to oceans and lakes as well as other adjacent urban domains, but these major coastal cities (e.g., Philadelphia, Boston, Miami, and New York) have sufficiently large reference areas (2,620 to 14,229 km^2^). The sensitivity test suggests that the urban signals are relatively stable for expanding reference domain (*SI Appendix*, section 7, Figs. S30–S32 and Table S1 for the sensitivity test). In addition to the spatial coverage difference, we also estimated the frequency of enhanced urban cloud occurrences in a given month over 18 y (*f*_+_). This term is calculated as the ratio of daily +*Δ*_*CloudCover*_ occurrences over the total days in a given month for 18 y in the day and night overpasses, respectively.

### Local and Regional Factors and Statistical Modeling.

We evaluated the nonlinear influences of city size (i.e., A in km^2^) measured in a log scale (log_10_*A*), urban–rural difference in surface heating (*ΔLST*), climate background (annual precipitation and mean annual temperature, *P* and $\bar{T}$) on the spatial variation of *Δ*_*CloudCover*_ on urban cloud cover anomalies. Only cities with significant *Δ*_*CloudCover*_ at *P* = 0.05 level are analyzed.

MODIS LST Daily Product (MOD11A1 L3 C6.1) at 1-km resolution during 2002 to 2020 is used to estimate the strength of differential surface heating caused by cities. LST is a straightforward measurement of outgoing long-wave radiation (56), which has been used as an indicator to capture the urban–rural heating differences (57). Due to the uneven cloud distribution over the urban-background domain, we consider only the clear-sky LST images across each domain (i.e., images with at least 98% clear-sky pixels covered over a certain domain). The 18-y clear-sky temporal composite LST is estimated in a given month, and then, we calculated the urban-background differences using the same spatial criteria for cloud cover estimation. Note that MODIS LST data from Terra satellites are at around 10:30 (Terra-Day) and 22:30 (Terra-Night) local solar time, which is about 2 h earlier than the cloud cover. Strong surface heating and urban–rural heating differences can facilitate the development of the planetary boundary layer (PBL) leading to cloud formation in a sequential process. A previous study has shown that the heating peaks in the late morning and clouds subsequently peak around 2 to 4 h later (9). As clouds do not immediately appear when the surface heating peaks, the LST and cloud observations are not simultaneously collected. The same assumption applies to nighttime clouds. From the 18-y observations, this sequence of clear morning (evening) followed by cloudy afternoon (mid-night) conditions is very common (on average, ∼93% chances of all cases starting with clear-sky morning) (*SI Appendix*, Fig. S33 and section S8). *ΔLST* is estimated using the same urban/background domain definitions with cloud pattern quantification and temporally aggregated at the monthly scale. Due to the limited availability of clear-sky LST images, 333 urban domains [inland (187), coast (56), and mountain (90)] have valid *ΔLST* for statistical analysis.

We use North America Daymet product (1 km, 2002 to 2020) to estimate the background moisture (i.e., annual cumulative precipitation *P*) and energy (i.e., mean daily temperature $\bar{T}$) availability for each urban domain. Daymet is an extensively validated gridded estimate of daily weather parameters derived from ground weather observations (58). *P* and $\bar{T}$ consider areal average over combined background and transient domain outside each city (*SI Appendix*, Fig. S27). The 18-y average of annual *P* and annual $\bar{T}$ are calculated to represent the climate background.

We statistically analyzed the separate and joint contributions of local and regional drivers to urban cloud signals across CONUS in GAMs. GAMs are a powerful and flexible statistical tool to model multiple covariates with nonlinear relationships by smoothing splines (59). The effect of city size is estimated using the logarithm of actual city size because there are many small cities as compared to few large cities which skew the distribution of city size (60). We consider the response variable *Δ*_*CloudCover*_ in the Gaussian distributions (Fig. 2*A*), and each independent variable was fitted using the thin plate regression splines. The main implication of GAMs is to disentangle the nonlinear coinfluences of different drivers on the spatial anomalies of cloud patterns among cities. In addition to quantifying the main effect of climate background on the urban clouds, we also consider their interactive effect, which is jointly considered as the full tensor product smooth (*te*) the combination of the main and their marginal interactive effects (59). Eq. **1** shows the general model structure. We used the fast restricted maximum likelihood (fREML) method (61) for the model fitting.

[1] $$\begin{array}{c} \begin{array}{cc} y & =\alpha +s\left(\log_{10}A\right)+s\left(\Delta LST\right)\\ & \;+s\left(latitude,longitude\right)+te\left(P,\bar{T}\right)+\epsilon , \end{array} \end{array}$$

where *y* is *Δ*_*CloudCover*_ across CONUS domain in a given month and overpass (day or night), *α* is the intercept, *ϵ* is the residual error, *s* is the function of smooth terms, and *te* is the function accounted for the combined effect of main and marginal tensor product interaction.

## Materials and Methods

### Study Domains.

We considered all CONUS cities with more than 50-thousand inhabitants based on US Census Topologically Integrated Geographic Encoding and Referencing (TIGER) 2010 Urban Area product, resulting in 481 cities. After merging the contiguous cities, together we identified a total of 447 urban domains (cities defined in this study). We used the static boundary that is close to the midpoint of 2002 to 2020, and the urbanization rate is on average about 1.1% within the 2010 urban boundaries during the entire studied period (*SI Appendix*, Fig. S29). To disentangle the potentially strong influences of mesoscale circulations (e.g., sea–land and mountain–valley breezes) on cloud formations, we classified these chosen cities into three regional categories: inland (217), coast (127), and mountain (103) (*SI Appendix*, Fig. S1*A*). Coastal cities are those located within 70 km from the shore that are potentially subjected to the dominant sea–land breeze influences (48, 49). Similar to the mesoscale flow assumption for the sea–land breeze, orography can exert persistent and strong circulations (48, 50) that interact with urban areas to further modify the local cloud patterns. Cities located on complex terrains are likely subjected to mountain–valley circulations. Thus, we identified these mountainous cities with a greater than 1,000-m elevation difference with the highest point within 70-km buffered surrounding areas. We used Global Multi-resolution Terrain Elevation Data 2010 (GMTED2010) to identify the elevation. Inland cities are the rest of the noncoastal and nonmountainous cities. CONUS spans a vast area covering a variety of regional climate types. Using Köppen-Geiger climate classification at the first level, the climate backgrounds for all studied urban domains are then grouped into three major categories: cold, temperate, and arid climates (22) (Fig. 3).

### Estimation of Urban-modified Cloud Patterns.

The subdaily cloud mask layer from MODIS cloud masks (MYD35_L2 C6.1) from 2002 to 2020 is used to identify the cloud patterns. Each overpass of the MODIS onboard Aqua often covers two-hour windows around 13:30 (Aqua-Day) and 1:30 (Aqua-Night) local solar time near the equator, offering the day and nighttime results, respectively. The cloud mask product is generated from a series of classic cloud screening algorithms (51) using a synergy of infrared emittance and visible reflectance, which is widely acceptable for developing other MODIS products and applications. The cloud screening accuracy and uncertainties have been extensively assessed (e.g., ref. 52). Potential biases are reported over ice-snow backgrounds and nighttime and mainly over high-latitude polar regions (53, 54). Our focus is on mid-latitude cities, which are not subject to the strong influences of ice-snow backgrounds. Here, we consider the pixel classified as probably cloudy or cloudy for cloud occurrence and cover in this study. Compared to the geostationary visible-band information for daytime-only analyses in the existing literature, using this relatively high-resolution and subdaily cloud cover largely improves the climatological understanding of diurnal and seasonal changes in cloud patterns at the city scale.

The urban-induced cloud pattern changes (*Δ*_*CloudCover*_) were quantified as the spatial differences of cloud coverage in percent between an urban area and its surrounding background. Cloud coverage is estimated as a fraction of cloudy areas identified by the cloud mask layer relative to the corresponding domain area. The daily *Δ*_*CloudCover*_ then were aggregated at a monthly scale over 18 y at each given urban domain. We have conducted the t test to identify the significant urban *Δ*_*CloudCover*_ signals for individual cities in each given month over 18 y at *p* = 0.05 level (*SI Appendix*, Figs. S3 and S4 and section S2 for the detail descriptions and quantitative results of the significance test). The urban areas are defined using US Census urban areas product, and the background is each urban domain’s surrounding region but excludes a transition buffered area to avoid urban-influenced clouds in upwind and downwind directions in the atmosphere. The transition and background areas are buffered proportional to the urban size (*SI Appendix*, Fig. S27 for the specific definition and an example of the transition zone and the background reference). Large water bodies (water bodies with an area larger than 1 km^2^) also have strong local effects on clouds (*SI Appendix*, Fig. S28), thus we excluded waterbodies across the urban-background domains to avoid nonurban influences (55). The nonurban reference can reach up to five times the urban area. There are about 6% of total studied cities that have a similar size or smaller reference domain due to proximity to oceans and lakes as well as other adjacent urban domains, but these major coastal cities (e.g., Philadelphia, Boston, Miami, and New York) have sufficiently large reference areas (2,620 to 14,229 km^2^). The sensitivity test suggests that the urban signals are relatively stable for expanding reference domain (*SI Appendix*, section 7, Figs. S30-S32 and Table S1 for the sensitivity test). In addition to the spatial coverage difference, we also estimated the frequency of enhanced urban cloud occurrences in a given month over 18 y (*f*_+_). This term is calculated as the ratio of daily +*Δ*_*CloudCover*_ occurrences over the total days in a given month for 18 y in the day and night overpasses, respectively.

### Local and Regional Factors and Statistical Modeling.

We evaluated the nonlinear influences of city size (i.e., A in km^2^) measured in a log scale (log_10_*A*), urban–rural difference in surface heating (*ΔLST*), climate background (annual precipitation and mean annual temperature, *P* and $\bar{T}$) on the spatial variation of *Δ*_*CloudCover*_ on urban cloud cover anomalies. Only cities with significant *Δ*_*CloudCover*_ at *P* = 0.05 level are analyzed.

MODIS LST Daily Product (MOD11A1 L3 C6.1) at 1-km resolution during 2002 to 2020 is used to estimate the strength of differential surface heating caused by cities. LST is a straightforward measurement of outgoing long-wave radiation (56), which has been used as an indicator to capture the urban–rural heating differences (57). Due to the uneven cloud distribution over the urban-background domain, we consider only the clear-sky LST images across each domain (i.e., images with at least 98% clear-sky pixels covered over a certain domain). The 18-y clear-sky temporal composite LST is estimated in a given month, and then, we calculated the urban-background differences using the same spatial criteria for cloud cover estimation. Note that MODIS LST data from Terra satellites are at around 10:30 (Terra-Day) and 22:30 (Terra-Night) local solar time, which is about 2 h earlier than the cloud cover. Strong surface heating and urban–rural heating differences can facilitate the development of the planetary boundary layer (PBL) leading to cloud formation in a sequential process. A previous study has shown the heating peaks in the late morning and clouds subsequently peak around 2 to 4 h later (9). Clouds do not immediately appear when the surface heating peaks; thus, the LST and cloud observations are not simultaneously collected. The same assumption applies to nighttime clouds. From the 18-y observations, this sequence of clear morning (evening) followed by cloudy afternoon (mid-night) conditions is very common (on average, ∼93% chances of all cases starting with clear-sky morning) (*SI Appendix*, Fig. S33 and section S8). *ΔLST* is estimated using the same urban/background domain definitions with cloud pattern quantification and temporally aggregated at the monthly scale. Due to the limited availability of clear-sky LST images, 333 urban domains [inland (187), coast (56), and mountain (90)] have valid *ΔLST* for statistical analysis.

We use North America Daymet product (1 km, 2002 to 2020) to estimate the background moisture (i.e., annual cumulative precipitation *P*) and energy (i.e., mean daily temperature $\bar{T}$) availability for each urban domain. Daymet is an extensively validated gridded estimate of daily weather parameters derived from ground weather observations (58). *P* and $\bar{T}$ consider areal average over combined background and transient domain outside each city, respectively (*SI Appendix*, Fig. S27). The 18-y average of annual *P* and annual $\bar{T}$ are calculated to represent the climate background.

We statistically analyzed the separate and joint contributions of local and regional drivers to urban cloud signals across CONUS in GAMs. GAMs are a powerful and flexible statistical tool to model multiple covariates with nonlinear relationships by smoothing splines (59). The effect of city size is estimated using the logarithm of actual city size because there are many small cities as compared to few large cities which skew the distribution of city size (60). We consider the response variable *Δ*_*CloudCover*_ in the Gaussian distributions (Fig. 2*A*), and each independent variable was fitted using the thin plate regression splines. The main implication of GAMs is to disentangle the nonlinear coinfluences of different drivers on the spatial anomalies of cloud patterns among cities. In addition to quantifying the main effect of climate background on the urban clouds, we also consider their interactive effect, which is jointly considered as the full tensor product smooth (*te*) the combination of the main and their marginal interactive effects (59). Eq. **2** shows the general model structure. We used the fast restricted maximum likelihood (fREML) method (61) for the model fitting.

[2] $$\begin{array}{c} \begin{array}{cc} y & =\alpha +s\left(\log_{10}A\right)+s\left(\Delta LST\right)\\ & \;+s\left(latitude,longitude\right)+te\left(P,\bar{T}\right)+\epsilon , \end{array} \end{array}$$

where *y* is *Δ*_*CloudCover*_ across the CONUS domain in a given month and overpass (day or night), *α* is the intercept, *ϵ* is the residual error, *s* is the function of smooth terms, and *te* is the function accounted for the combined effect of main and marginal tensor product interaction.

## Supplementary Material

Appendix 01 (PDF)

## Data Availability

Population are from US Census TIGER 2010 Urban Area product: https://catalog.data.gov/dataset/tiger-line-shapefile-2018-2010-nation-u-s-2010-census-urban-area-national. The coastlines are from the US Census TIGER 2019 Coastline product: https://catalog.data.gov/dataset/tiger-line-shapefile-2019-nation-u-s-coastline-national-shapefile. Elevation is from GMTED2010 product: https://www.usgs.gov/centers/eros/science/usgs-eros-archive-digital-elevation-global-multi-resolution-terrain-elevation. MODIS cloud masks (MYD35_L2 C6.1) and LST products (MOD11A1 L3 C6.1) can be downloaded from The Level-1 and Atmosphere Archive & Distribution System Distributed Active Archive Center (LAADS DAAC): https://ladsweb.modaps.eosdis.nasa.gov/ (accessed on 29 September 2022). Daymet product is publicly available at The Oak Ridge National Laboratory Distributed Active Archive Center (ORNL DAAC): https://daac.ornl.gov/about/. Data used for the estimation of the role of contributing factors on urban local cloud patterns (GAMs analysis) are deposited at https://doi.org/10.5281/zenodo.7130246 (62).
