# Supplementary material for: Urban effects on local cloud patterns
Source: Proc Natl Acad Sci U S A. 2023 May 15;120(21):e2216765120. doi: 10.1073/pnas.2216765120 (PMC10214127; doi:10.1073/pnas.2216765120)
Supplement: Supplementary file 1 — Appendix 01 (PDF) [file pnas.2216765120.sapp.pdf]

PNAS

Supporting Information for

Urban Effects on Local Cloud Patterns

Thuy Trang Vo[1], Leiqiu Hu[1,*], Lulin Xue[2], Qi Li[3], and Sisi Chen[2]

[1] Department of Atmospheric and Earth Science, The University of Alabama in Huntsville, Huntsville, AL, USA
[2] Research Applications Laboratory, National Center for Atmospheric Research, Boulder, CO, USA
[3] School of Civil and Environmental Engineering, Cornell University, Ithaca, NY, USA

*Corresponding author: Leiqiu Hu
Email: leiqiu.hu@uah.edu

This PDF file includes:

Figure S1 to Figure S33
SI References
Section S1-S8
Table S1

Section S1
Observational summary (seasonal, diurnal, and spatial analyses)

a. Study domain

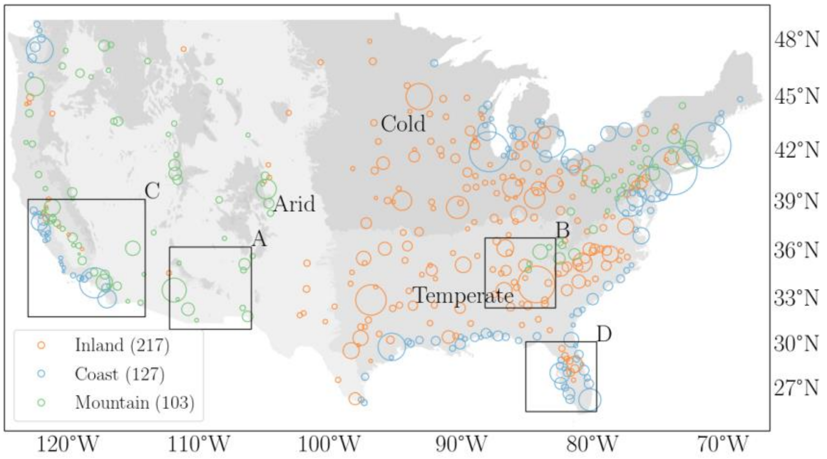

b. 18-year monthly composite cloud occurrences (frequency of cloudy pixels) at four example sub regions (July, Day)

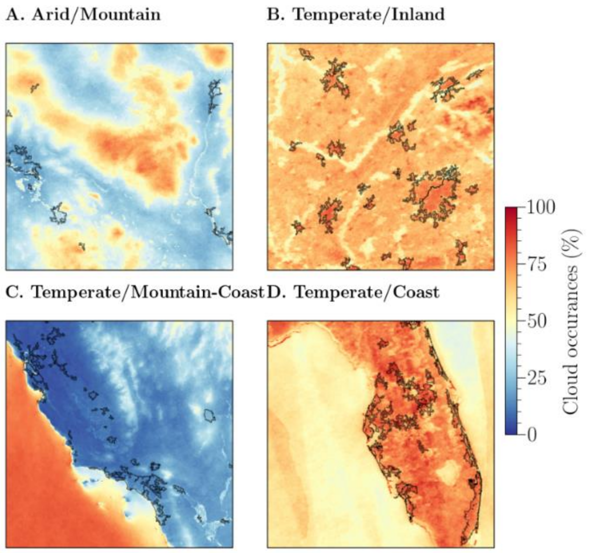

Figure S1. 447 CONUS cities are categorized based on geographical context: inland, coast and mountain. (a) Three types of climate background are shown in different shaded color in the base map. Each circle represents for a city, and its size is proportional to the city size. The total numbers of cities in each category are labeled in the map. (b) Examples of monthly composite cloud occurrences across four regions (A-D specified in (a)) in CONUS in July (Aqua-day; ~1 pm).

. Daytime

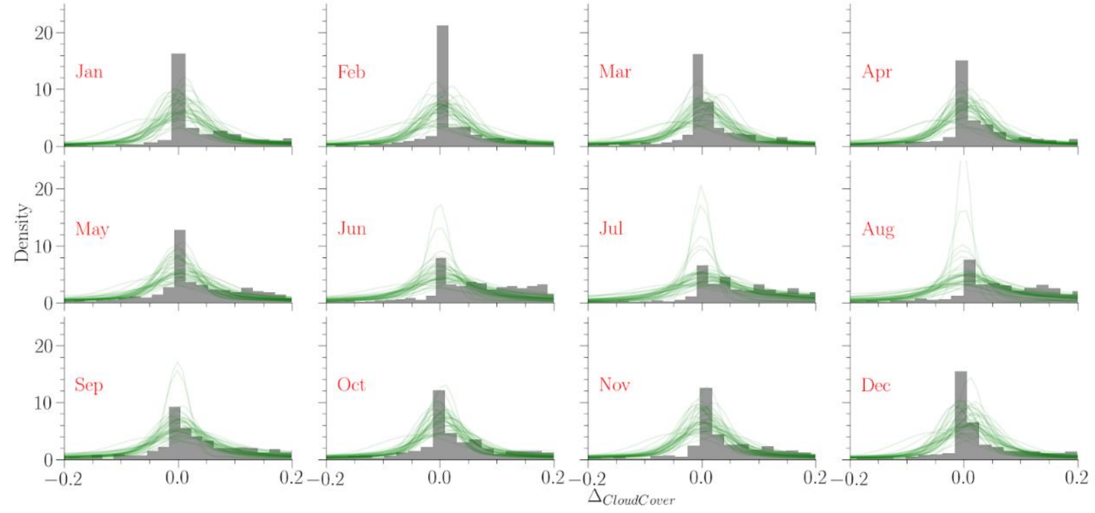

b. Nighttime

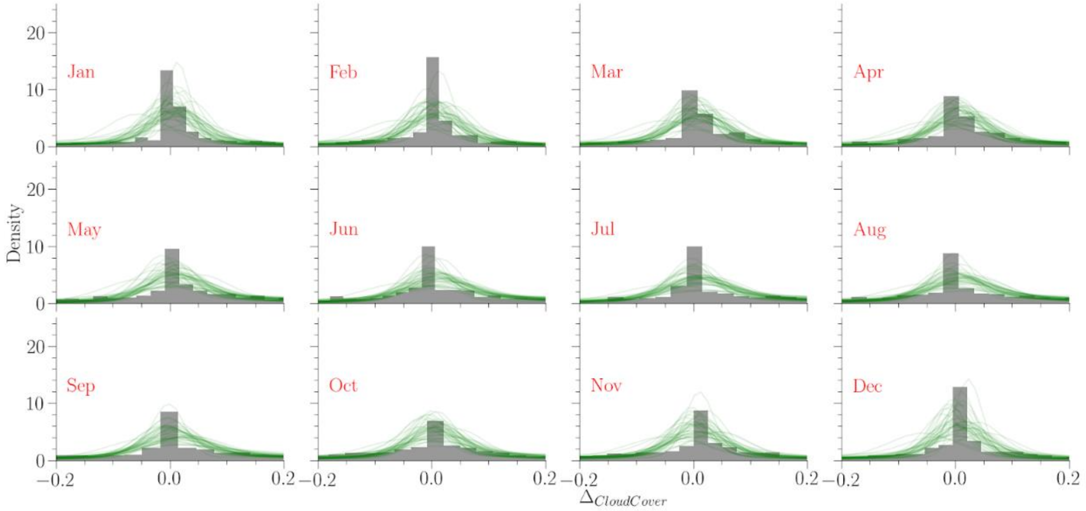

Figure S2. Probability density function of daily cloud spatial anomalies ($\Delta_{\text{CloudCover}}$) for randomly selected 40 cities as green fitted lines and Houston, TX shown in the gray histogram.

Section S2
Significance test for ΔCloudCover

ΔCloudCover is estimated at the daily scale, and then we aggregated the daily values to the monthly scale. The cloud cover contrast is a simultaneous estimate for each city. In each month period (31 days x 18 years), the daily values follow a bell-shape distribution over the 18-year period (see **Figure S2**). We used one sample two tailed t-test to identify whether the mean of ΔCloudCover at each city in each month (31 days x 18 years) is different from zero. Majority of cities (63.5 - 90.2 % of cities among day/night and different months) show statistically significant results at p=0.05 level (**Figure S4**). **Figure S3** shows the distributions of means, upper and lower bounds of the 95% confidence intervals across all studied cities. The urban effects on cloud patterns (manuscript used approach) have the confidence level (upper and lower limits) falls above zero for summer months, particularly at night. For months with strong nighttime reduction (such as winter nighttime), the lower confidence bound suggest mostly negative values.

(a) Distribution of ΔCloudCover (%) of all studied cities (identical to Fig. 2a in main manuscript)

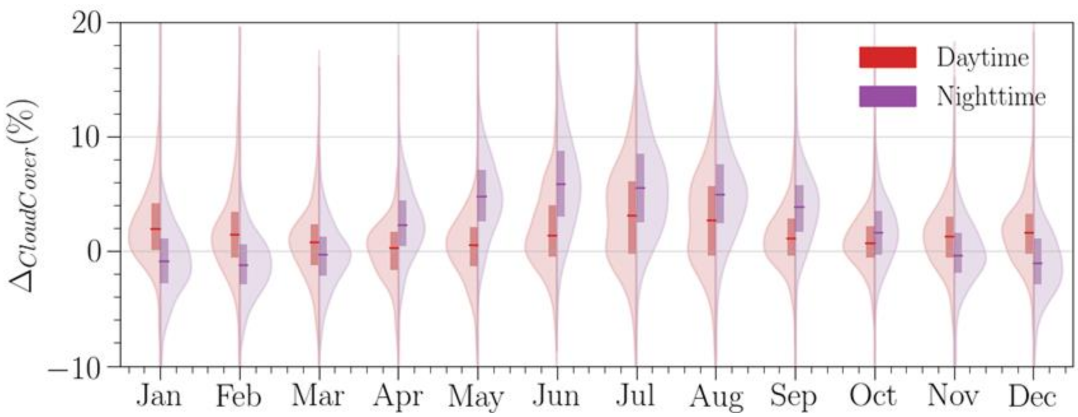

(b) Upper limit of the 95% confidence interval from t-test results

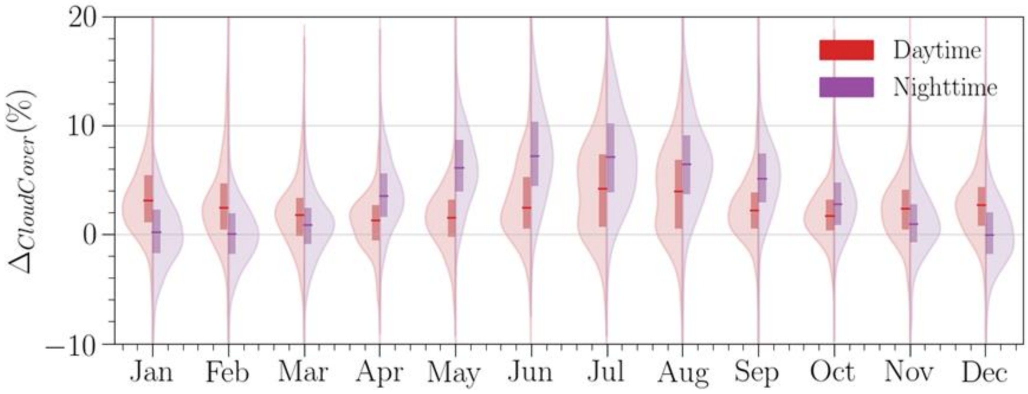

(c) Lower limit of the 95% confidence interval from t-test results

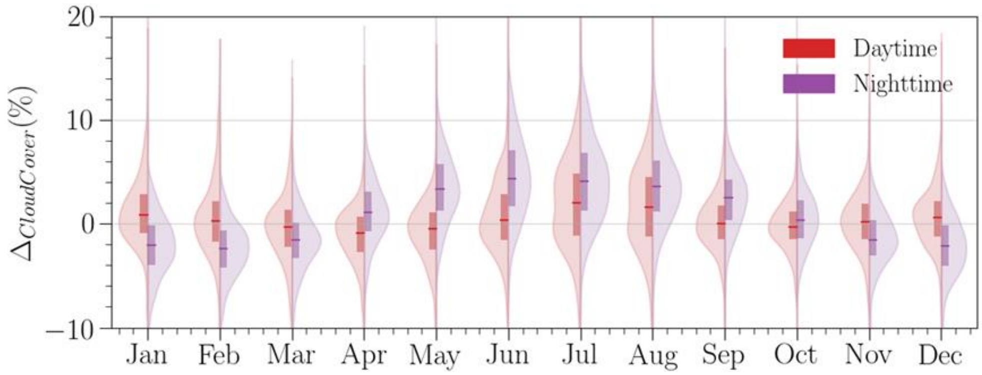

Figure S3. The results from t-test estimated mean (a), upper (b) and (c) lower limits of the 95% confidence intervals.

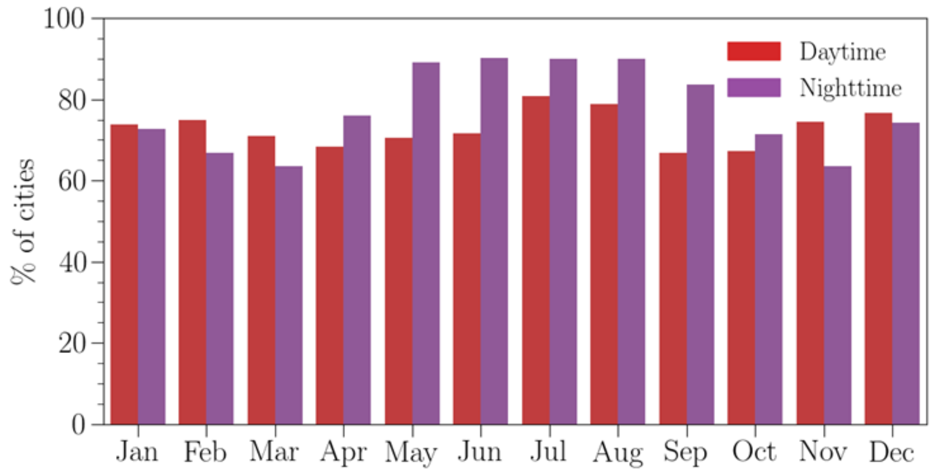

Figure S4. Characterization of percentage of cities showing the statistically significant results of t test (at p= 0.05 level) for ΔCloudCover.

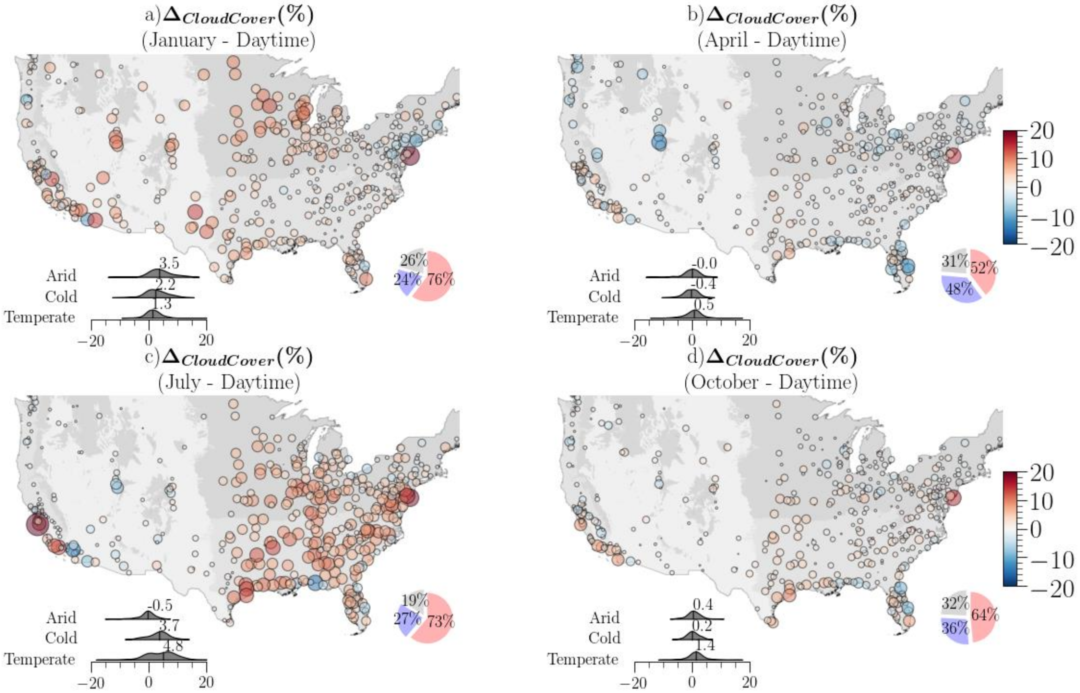

Figure S5. Spatial variations of urban modified cloud cover ($\Delta_{CloudCover}$) across CONUS during the daytime in January (a), April (b), July (c) and October (d). Each circle represents a city. Its color indicates the intensity of the estimated variable, and its size is proportional to $|\Delta_{CloudCover}|$. A distribution comparison among three climate regions is shown at the left corner as density curves and the median value for each region is labeled. The ratios of studied cities with $+\Delta_{CloudCover}$ are shown in red and $-\Delta_{CloudCover}$ in blue, and are summarized in the pie plot at the lower right corner. The ratios of studied cities with insignificant results are shown in grey.

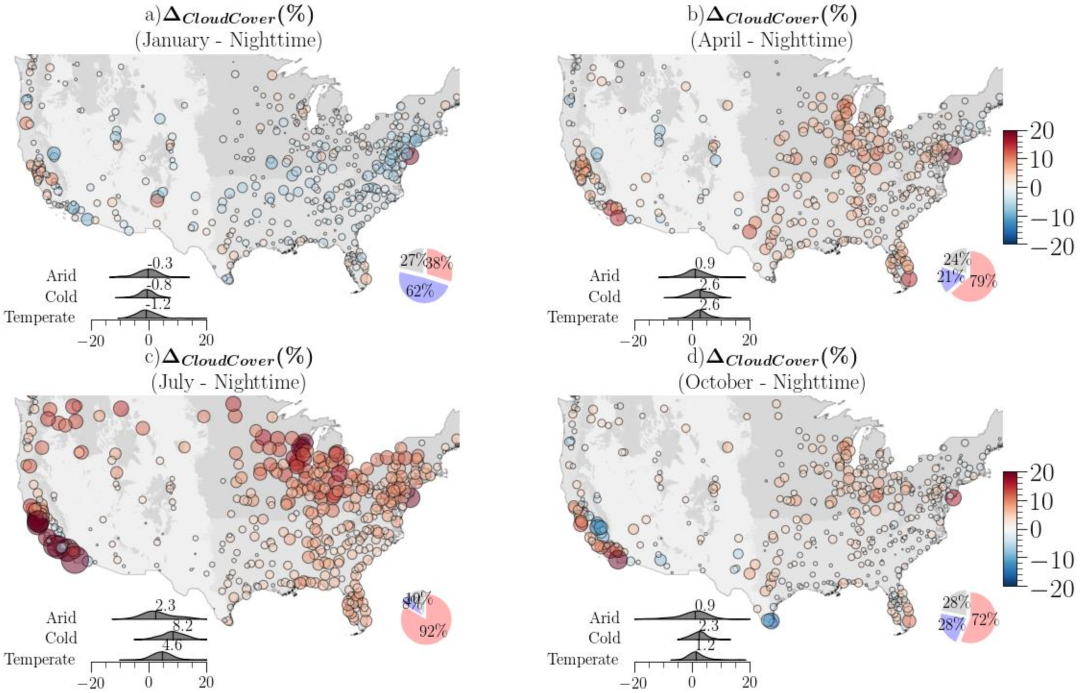

Figure S6. Spatial variations of urban modified cloud cover ($\Delta_{CloudCover}$) across CONUS during the nighttime in January (a), April (b), July (c) and October (d). Each circle represents a city. Its color

indicates the intensity of the estimated variable, and its size is proportional to $|\Delta_{CloudCover}|$. A distribution comparison among three climate regions is shown at the left corner as density curves and the median value for each region is labeled. The ratios of studied cities with $+\Delta_{CloudCover}$ are shown in red and $-\Delta_{CloudCover}$ in blue, and are summarized in the pie plot at the lower right corner. The ratios of studied cities with insignificant results are shown in grey.

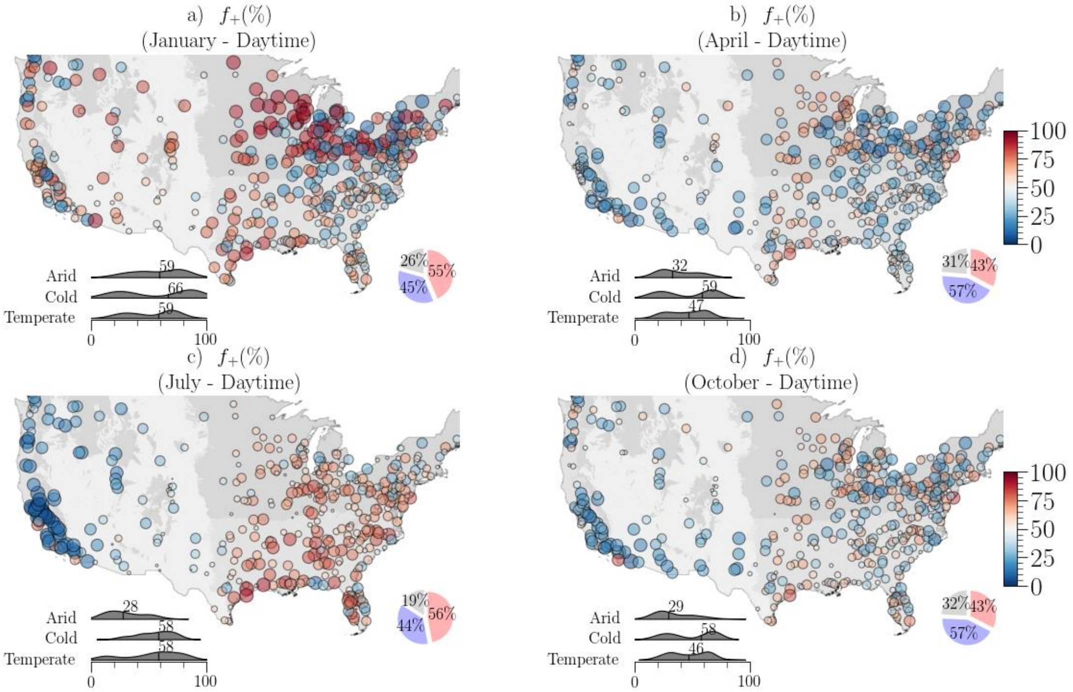

Figure S7. Spatial variations of urban modified cloud frequencies (f_+) across CONUS during the daytime in January (a), April (b), July (c) and October (d). Each circle represents a city. Its color indicates the intensity of the estimated variable, and its size is proportional to $|f_+ - 50\%|$. A distribution comparison among three climate regions is shown at the left corner as density curves and the median value for each region is labeled. The ratios of studied cities with $f_+ >= 50\%$ are shown in red and $f_+ < 50\%$ in blue, and are summarized in the pie plot at the lower right corner. The ratios of studied cities with insignificant results are shown in grey.

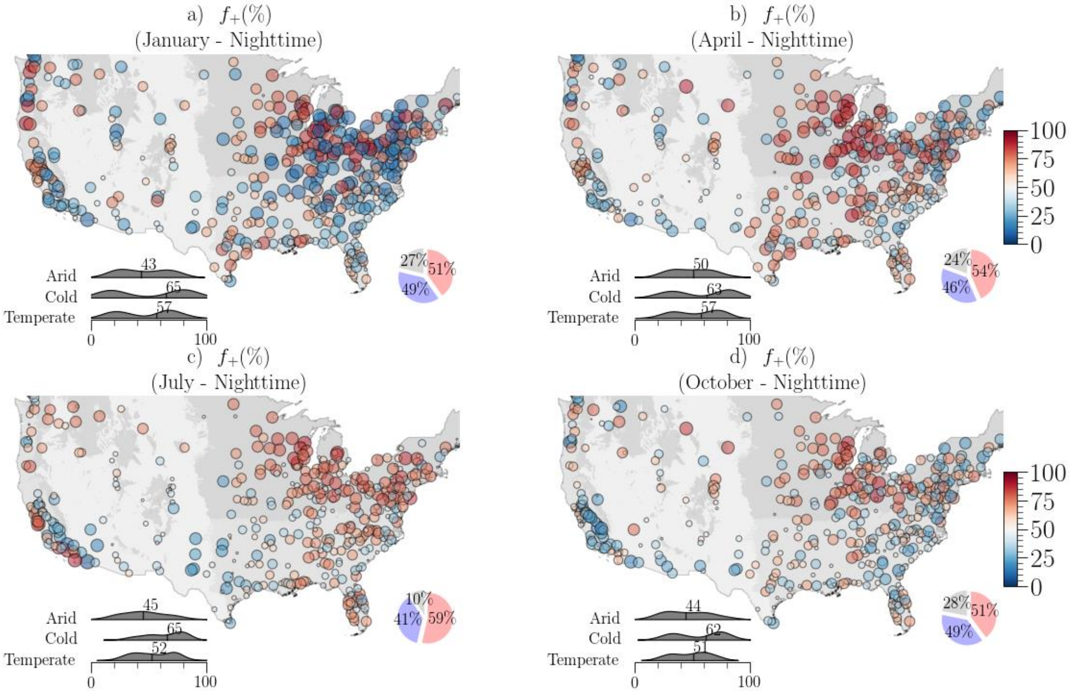

Figure S8. Spatial variations of urban modified cloud frequencies (f_+) across CONUS during the nighttime in January (a), April (b), July (c) and October (d). Each circle represents a city. Its color indicates the intensity of the estimated variable, and its size is proportional to $|f_+ - 50\%|$. A distribution comparison among three climate regions is shown at the left corner as density curves and the median value for each region is labeled. The ratios of studied cities with $f_+ >= 50\%$ are shown in red and $f_+ < 50\%$ in blue, and are summarized in the pie plot at the lower right corner. The ratios of studied cities with insignificant results are shown in grey.

Section S3
Attribution of local and regional factors on urban cloud patterns (GAMs summary)

Inland model. Cities located further than 70km from the shore and over flat-terrain regions are considered in the series of modeling that have limited impact from sea/land and mountain-valley breezes. The coastal and mountainous categories are described in the Methods. The separate and joint effects of city size (**Figure S10**), surface heating (**Figure S11**) and annual precipitation and mean air temperature (**Figure S12**) are quantified. The coefficient of determination (R^2) values for each month of the inland model are summarized as follows:

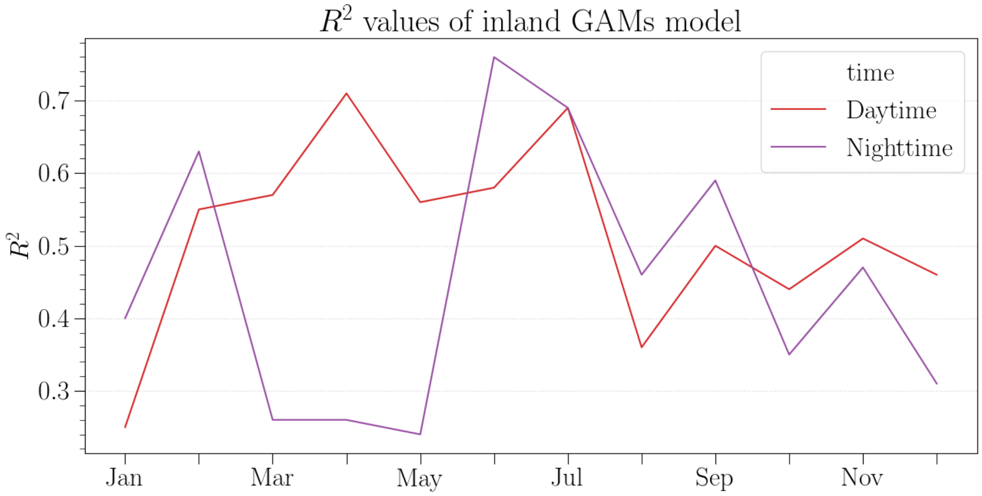

Figure S9. R^2 of models for inland cities

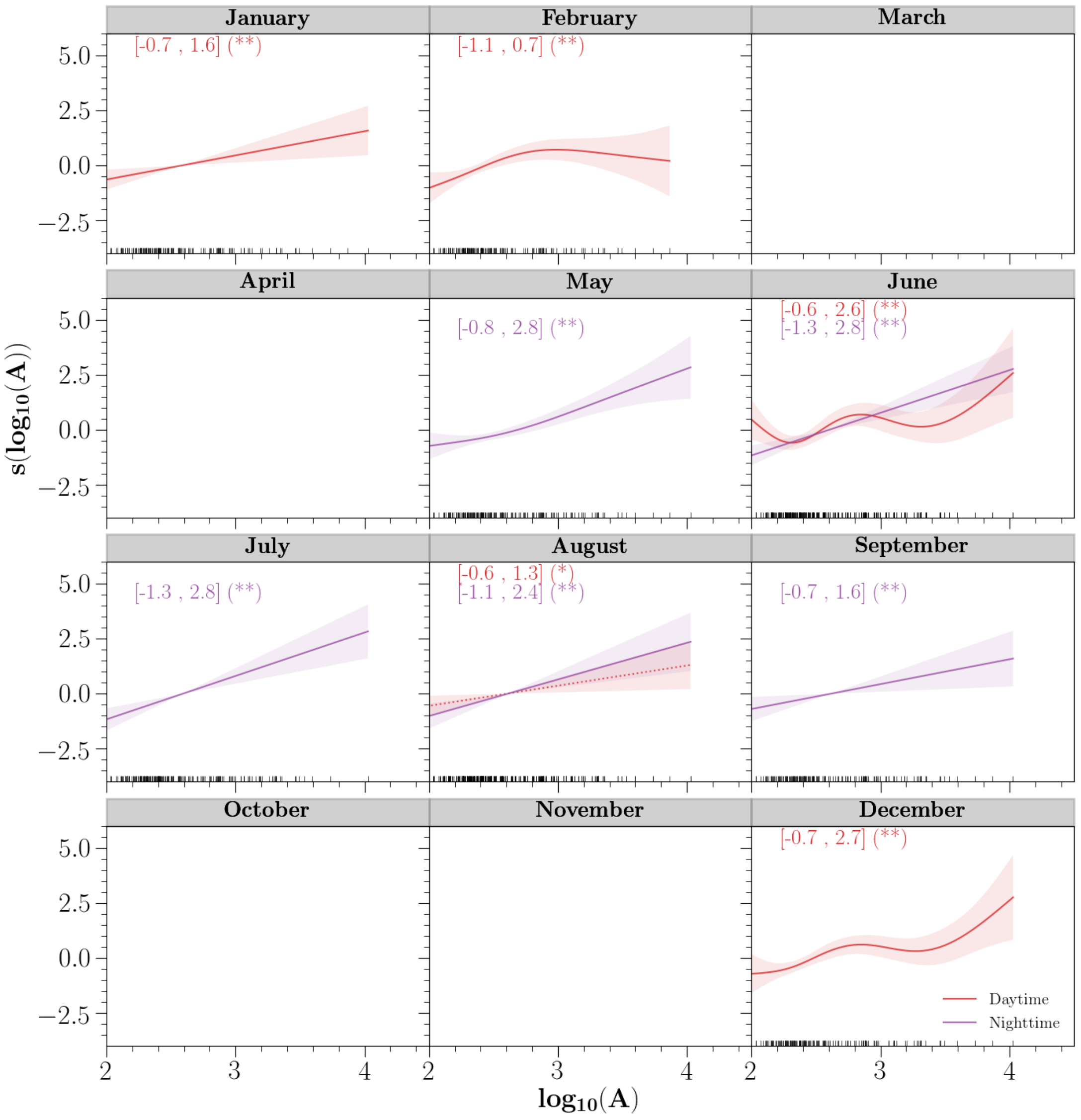

Figure S10. The main effect of city size in log scale ($\log_{10}A$, in $\log_{10}(km^2)$) on local cloud patterns ($\Delta_{CloudCover}$) in GAMs for inland cities from January to December. Only statistically significant results are illustrated and dashed lines suggested a p value at 0.05 level (*) and solid lines suggested p values at 0.01 level (**). The shaded regions depict the 2 standard errors of $\Delta_{CloudCover}$ proportion influenced by the logarithm of city size. The numbers in brackets are the minimum and maximum estimated effects associated with the denoted symbols of the statistically significant level. The vertical black lines are shown for the distribution of city size. The largest inland city, e.g., Atlanta, Georgia ($\log_{10}A = 4.02$) and the smallest inland city, e.g., Manhattan, Kansas ($\log_{10}A = 1.95$) are considered. Only cities with significant $\Delta_{CloudCover}$ are analyzed.

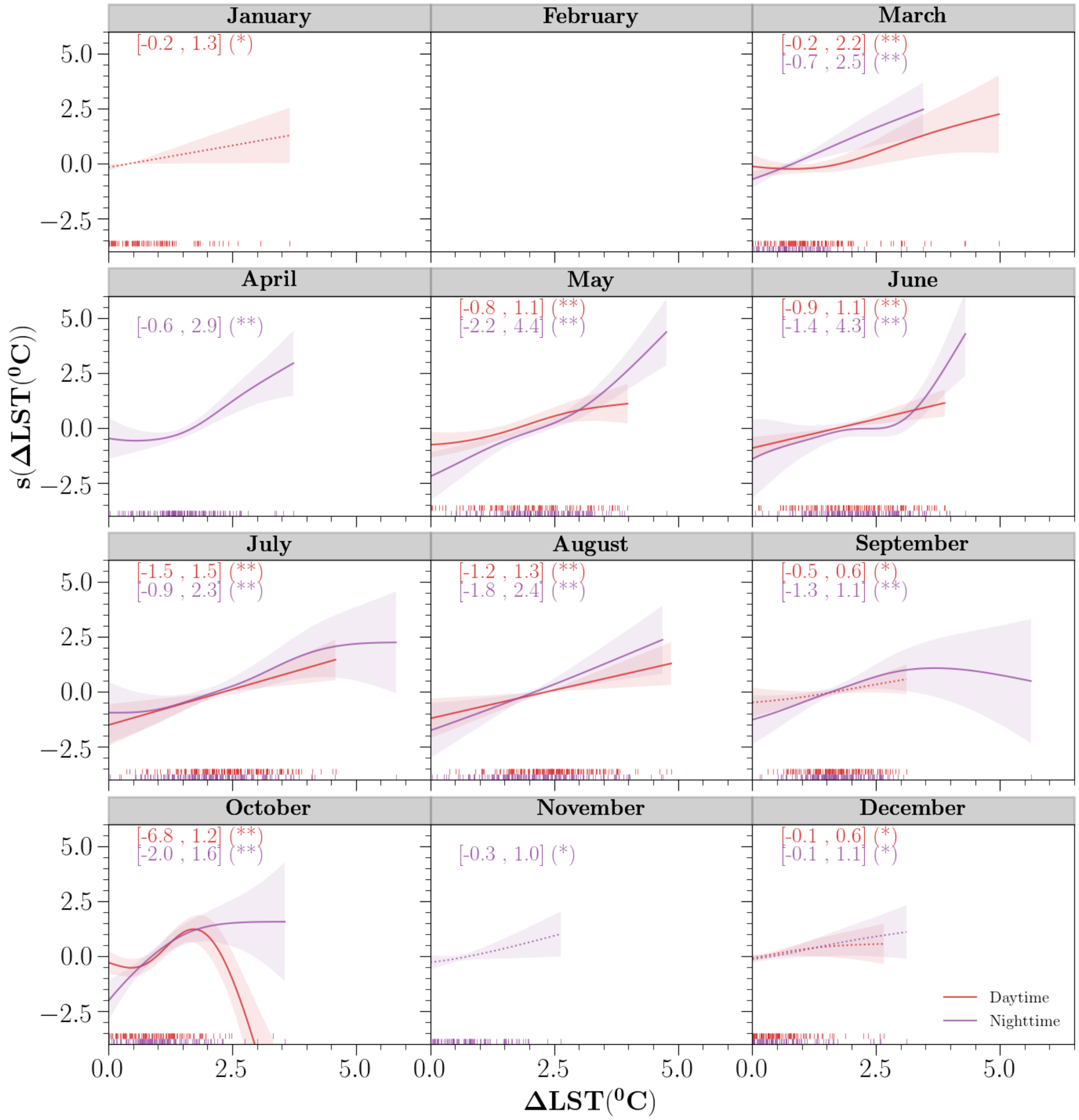

Figure S11. The main effect of surface heating (ΔLST, in ºC) on local cloud patterns ($\Delta_{\text{CloudCover}}$) in GAMs for inland cities from January to December. Only statistically significant results are illustrated and dashed lines suggested a p value at 0.05 level (*) and solid lines suggested p values at 0.01 level (**). The shaded regions depict the 2 standard errors of $\Delta_{\text{CloudCover}}$ proportion influenced by the surface heating. The numbers in brackets are the minimum and maximum estimated effects associated with the denoted symbols of the statistically significant level. The vertical lines are shown for the distribution of surface heating (in red and purple). Even though the statistical model includes also the negative ΔLST values, the estimated trends are not shown in this case. Only cities with significant $\Delta_{\text{CloudCover}}$ are analyzed.

a) Daytime

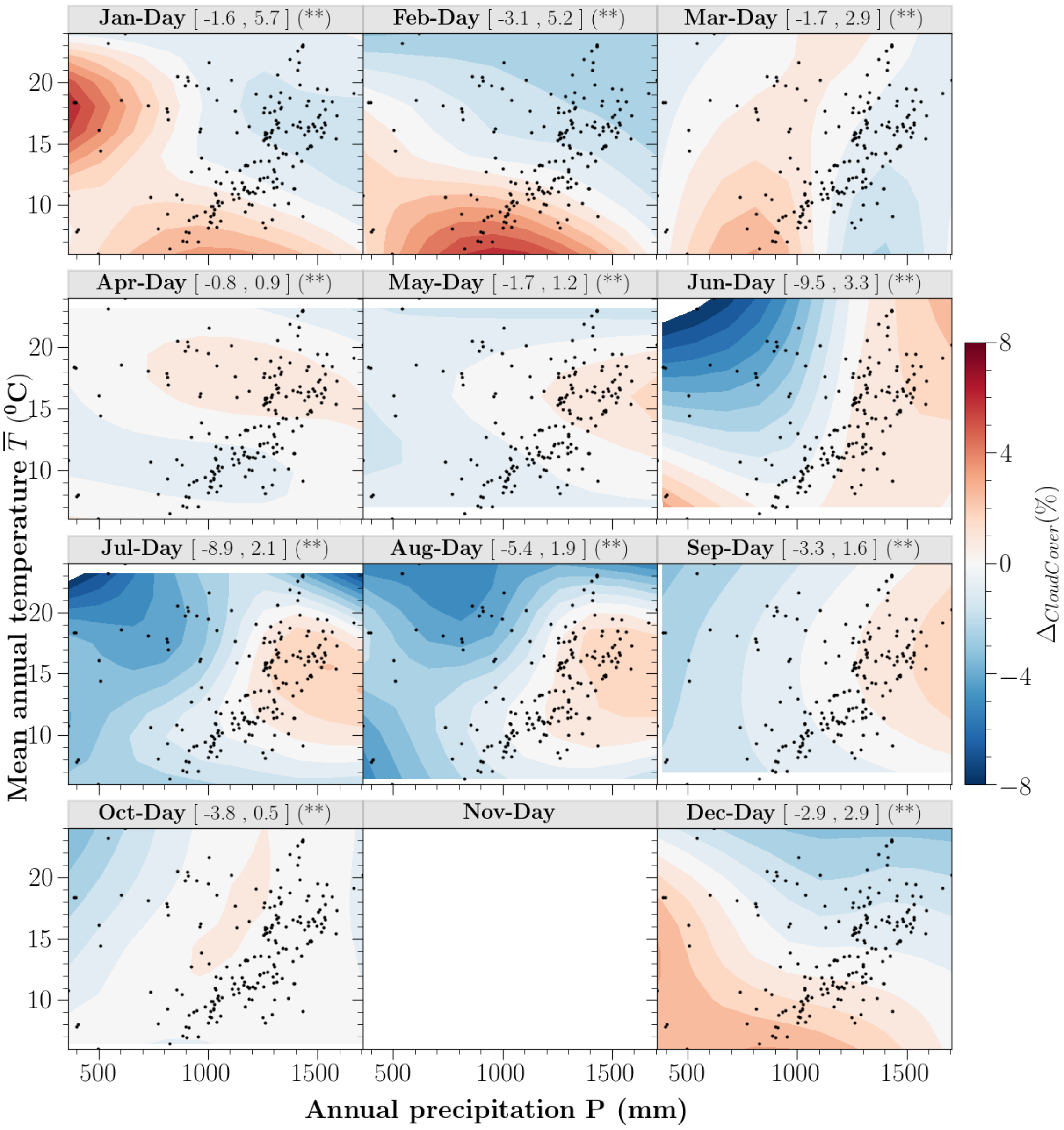

Jan-Day [-1.6 , 5.7] (**)
Feb-Day [-3.1 , 5.2] (**)
Mar-Day [-1.7 , 2.9] (**)
Apr-Day [-0.8 , 0.9] (**)
May-Day [-1.7 , 1.2] (**)
Jun-Day [-9.5 , 3.3] (**)
Jul-Day [-8.9 , 2.1] (**)
Aug-Day [-5.4 , 1.9] (**)
Sep-Day [-3.3 , 1.6] (**)
Oct-Day [-3.8 , 0.5] (**)
Nov-Day
Dec-Day [-2.9 , 2.9] (**)
Mean annual temperature $\overline{T}$ (^{0}C)
Annual precipitation P (mm)
$\Delta_{CloudCover}(\%)$

b) Nighttime

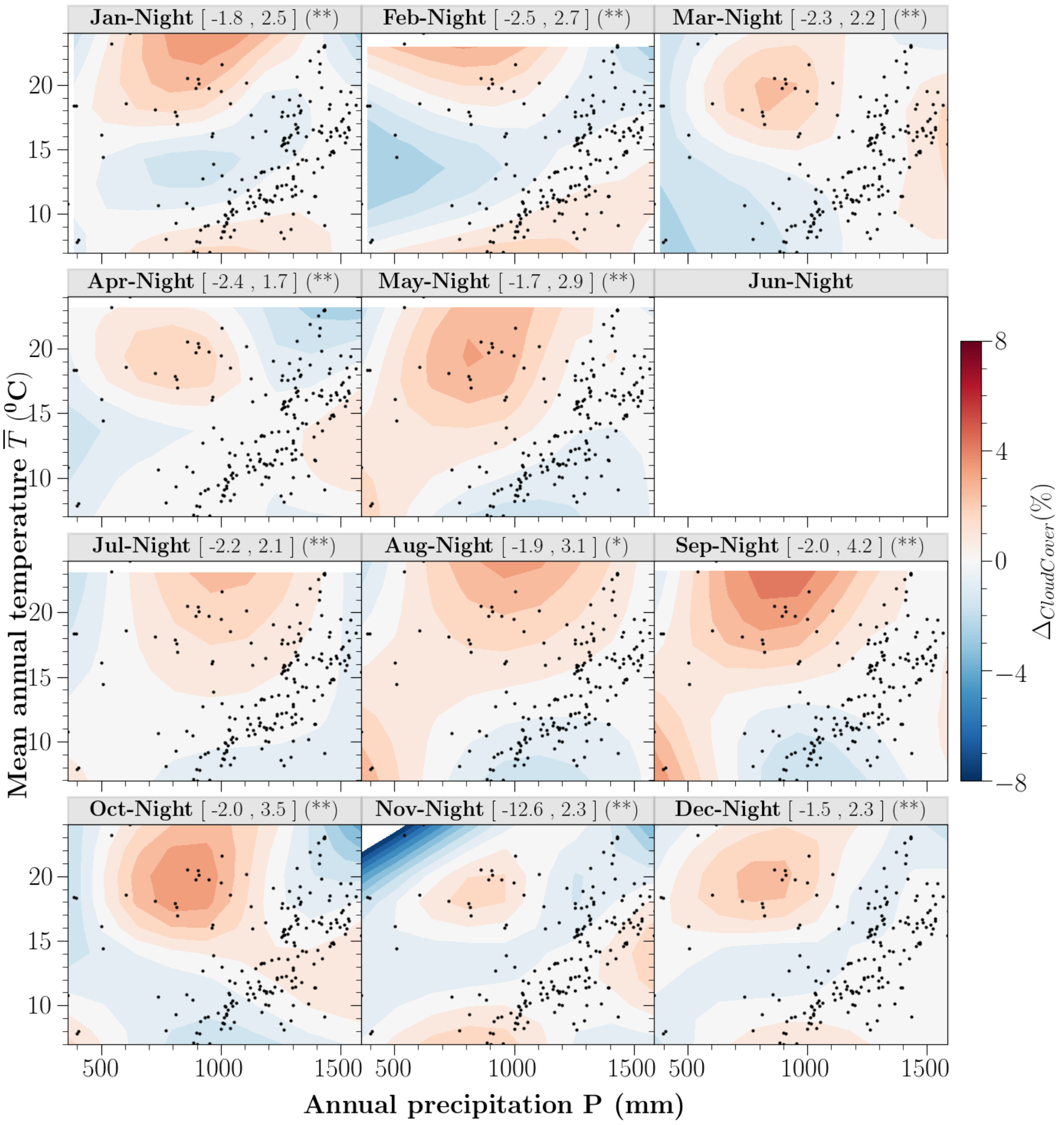

Figure S12. The combined effect (main + interactive effects) of **climate background (**annual precipitation P, in mm and mean annual temperature $\overline{\boldsymbol{T}}$, in °C on local cloud patterns ($\Delta_{\text{CloudCover}}$) in GAMs for **inland** cities from January to December, for daytime (a) and nighttime (b). Only statistically significant results are illustrated suggested a p value at 0.05 level (*) and at 0.01 level (**). The numbers in brackets are the minimum and maximum estimated effects associated with the denoted symbols of the statistically significant level. Only cities with significant $\Delta_{\text{CloudCover}}$ are analyzed.

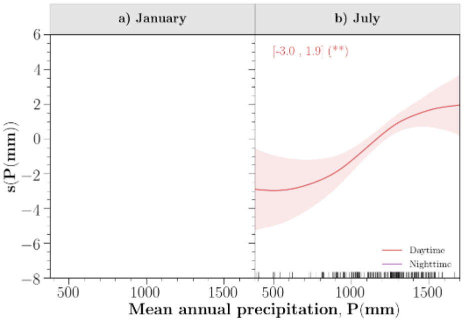

Figure S13. The main effect of moisture availability (annual precipitation P, in mm) on local cloud patterns ($\Delta_{CloudCover}$) in GAMs for inland cities in January (a) and July (b). Only statistically significant results are illustrated and dashed lines suggested a p value at 0.05 level (*) and solid lines suggested p values at 0.01 level (**). The shaded regions depict the 2 standard errors of $\Delta_{CloudCover}$ proportion influenced by the moisture availability. The numbers in brackets are the minimum and maximum estimated effects associated with the denoted symbols of the statistically significant level. The vertical lines are shown for the distribution of moisture availability for chosen cities.

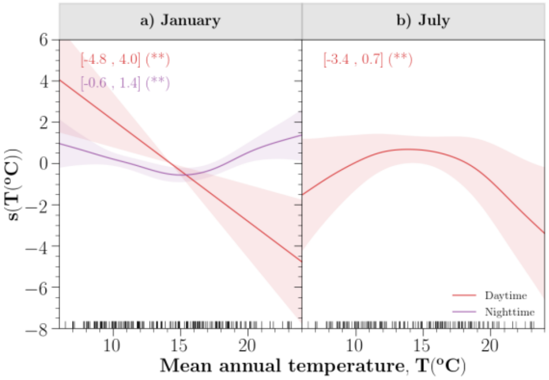

Figure S14. The main effect of energy availability (mean annual temperature $\overline{T}$, in °C) on local cloud patterns ($\Delta_{CloudCover}$) in GAMs for inland cities in January (a) and July (b). Only statistically significant results are illustrated and dashed lines suggested a p value at 0.05 level (*) and solid lines suggested p values at 0.01 level (**). The shaded regions depict the 2 standard errors of $\Delta_{CloudCover}$ proportion influenced by the energy availability. The numbers in brackets are the minimum and maximum estimated effects associated with the denoted symbols of the statistically significant level. The vertical lines are shown for the distribution of energy availability for chosen cities.

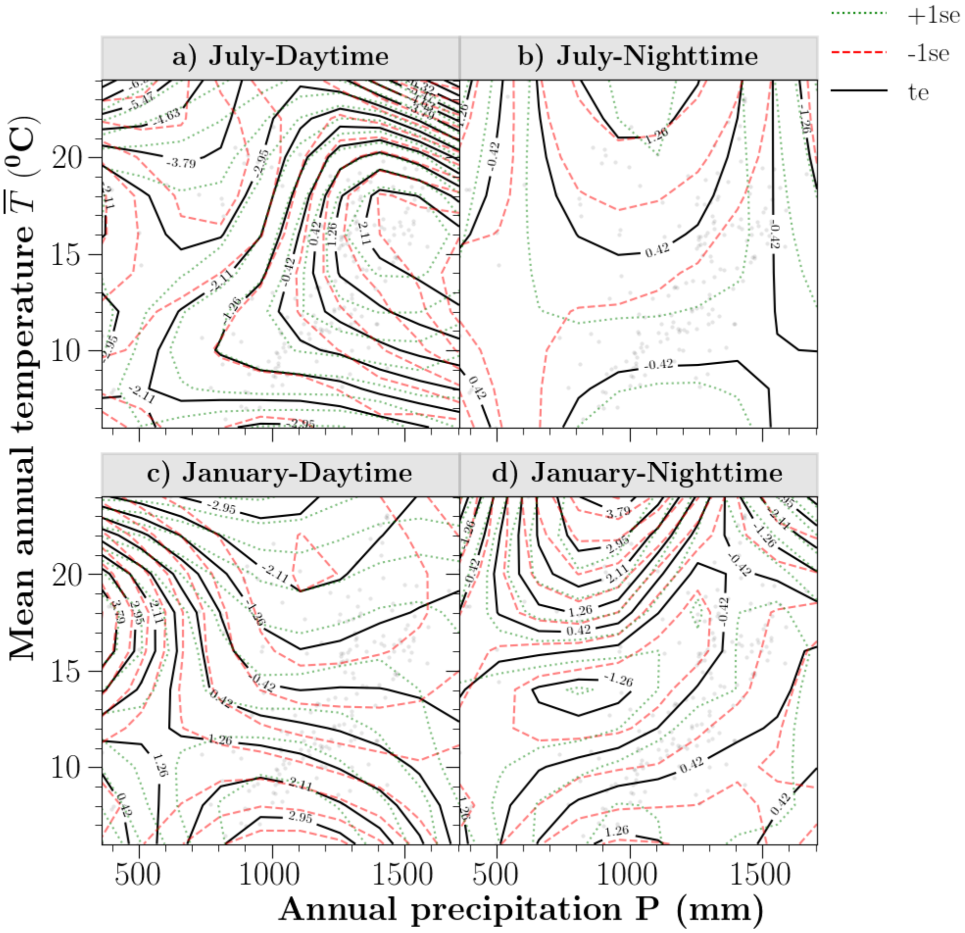

Figure S15. The combined effect (main + interactive effects) of climate background (annual precipitation P, in mm and mean annual temperature $\overline{T}$ in ^{o}C on local cloud patterns ($\Delta_{\mathrm{CloudCover}}$) in GAMs for inland cities in July and January for day and night. Black solid lines suggest the estimated effect (labeled as te) of the climate background on local cloud patterns and labeled accordingly. Red and green dashed lines indicate the -1 and +1 standard errors (se) of the estimated effects, respectively.

Coastal model. As the coastal and mountainous cities are facing more complex mesoscale influences, the analysis is built separately for coastal and mountainous cities. Coastal cities are those located within 70 km from the shore that are potentially subject to the dominant sea-land breeze influences (Thunis 1996, Kusaka 2019).

Based on the definition of U.S. coastal regions, coastal cities are also divided into four main U.S. coastal classification: Pacific Coast, Atlantic Coast, Gulf Coast and Great Lakes. We observe a much larger variation of summer urban cloud changes among different coastal cities (**Figure S17**).

The coastal and mountainous categories are described in the Materials and Methods. The separate and joint effects of city size (**Figure S18**), surface heating (**Figure S19**) and annual precipitation and mean air temperature (**Figure S20**) are quantified. The coefficient of determination (R^2) values for each month of are summarized as follows:

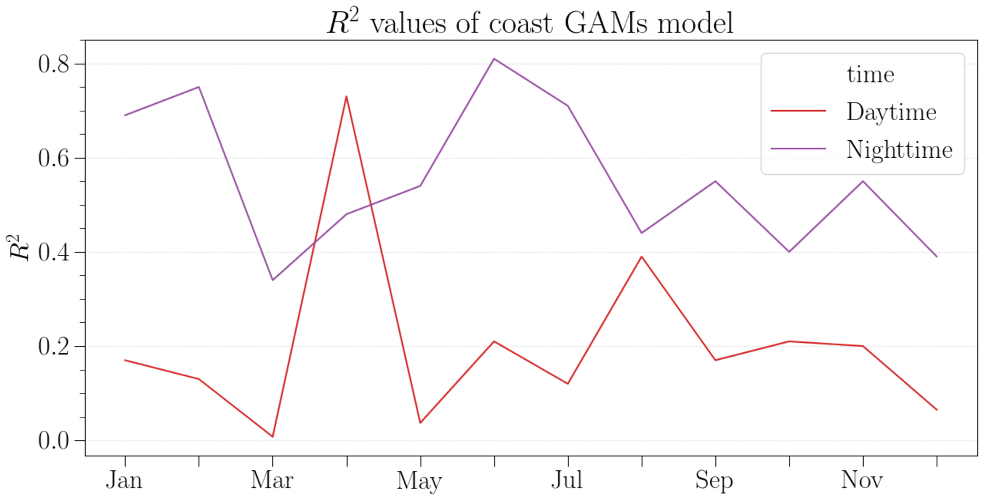

Figure S16. R^2 of models for coastal cities

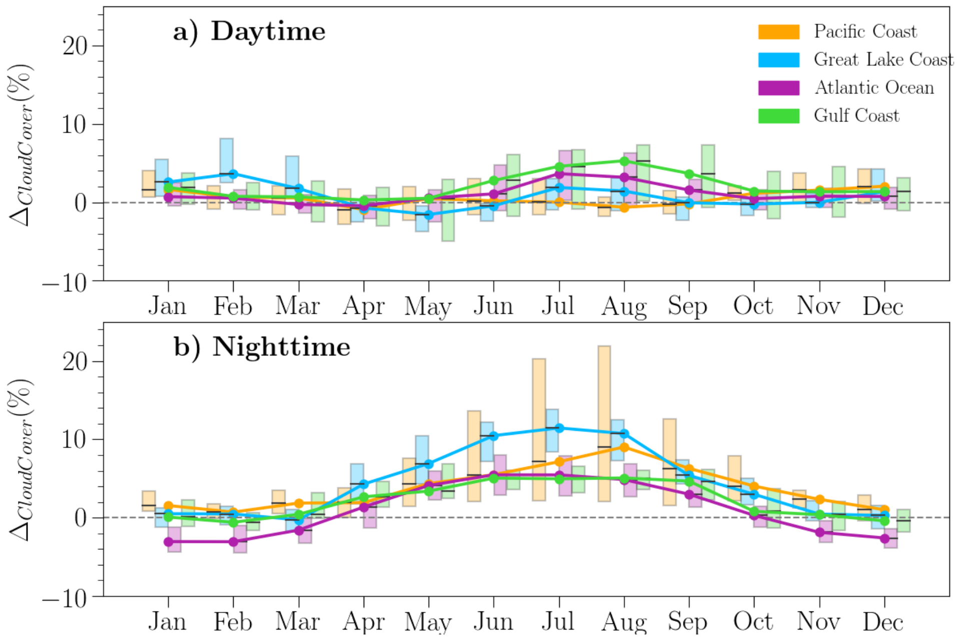

Figure S17. Seasonal and diurnal variations of $\Delta_{\mathrm{CloudCover}}$ across different coastal regions. (a) Daytime, and (b) Nighttime. Boxes show the median (solid line) and interquartile range (IQR) of the $\Delta_{\mathrm{CloudCover}}$.

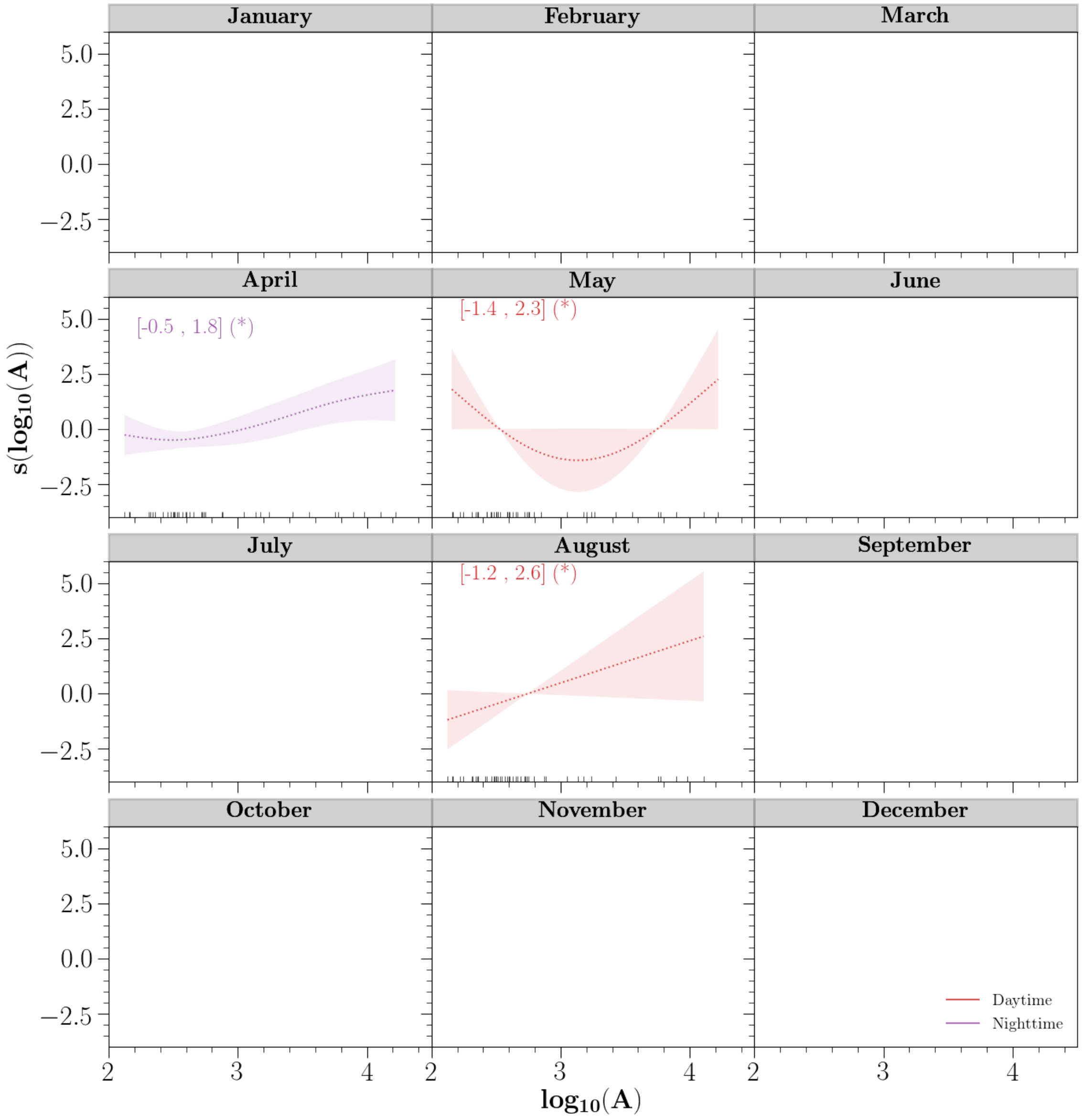

Figure S18. The main effect of city size in log scale ($\log_{10}A$, in $\log_{10}(km^2)$) on local cloud patterns ($\Delta_{CloudCover}$) in GAMs for coastal cities from January to December. Only statistically significant results are illustrated and dashed lines suggested a p value at 0.05 level (*) and solid lines suggested p values at 0.01 level (**). The shaded regions depict the 2 standard errors of $\Delta_{CloudCover}$ proportion influenced by the logarithm of city size. The numbers in brackets are the minimum and maximum estimated effects associated with the denoted symbols of the statistically significant level. The vertical black lines are shown for the distribution of city size. The largest coastal city, e.g., Boston, Massachusetts - New Hampshire - Rhode Island ($\log_{10}A = 4.2$) and the smallest coastal city, e.g., Pascagoula, Mississippi ($\log_{10}A = 2.1$) are considered. Only cities with significant ΔCloudCover at p = 0.05 level are analyzed.

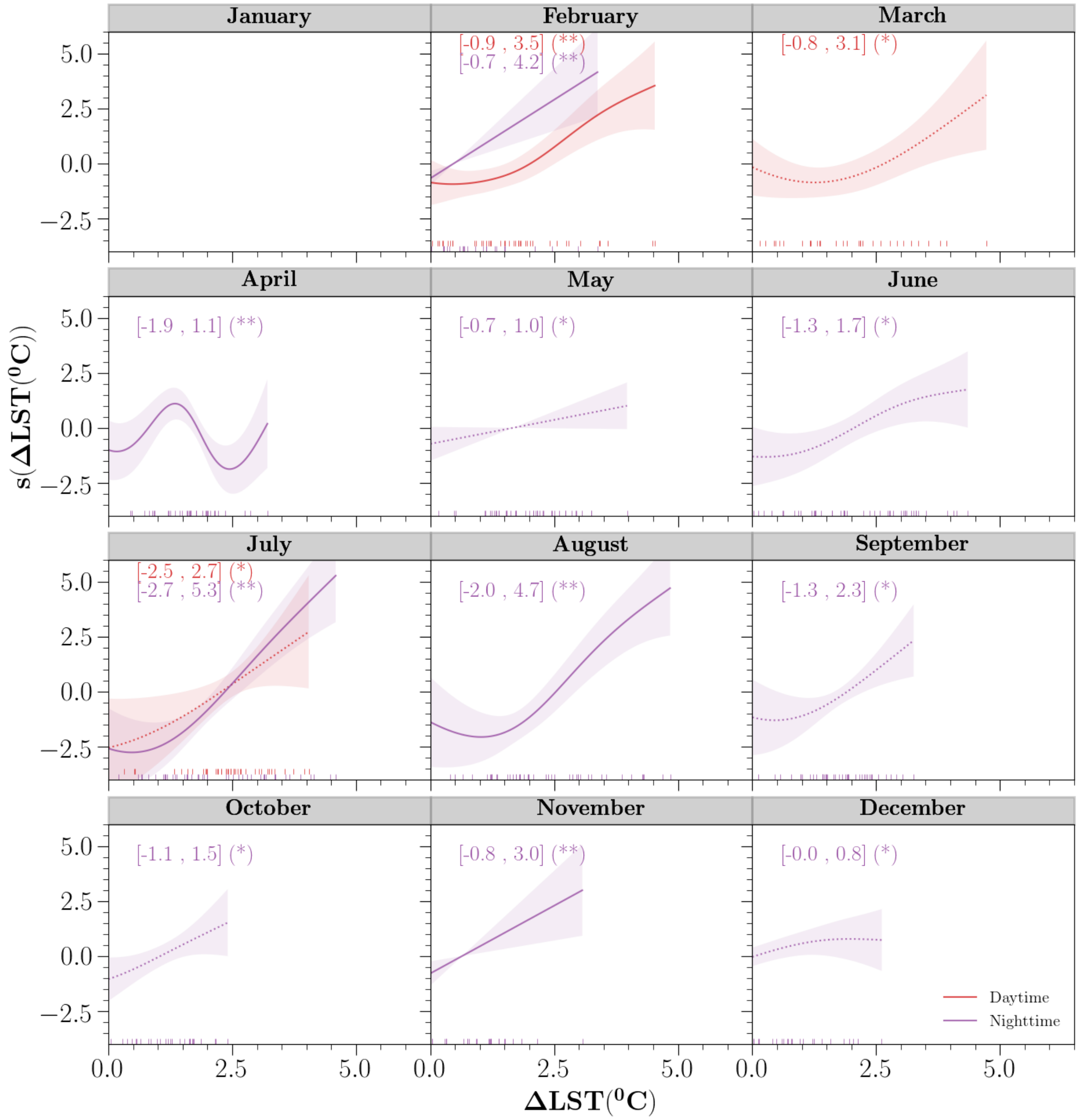

Figure S19. The main effect of surface heating (ΔLST, in ºC) on local cloud patterns ($\Delta_{\text{CloudCover}}$) in GAMs for coastal cities from January to December. Only statistically significant results are illustrated and dashed lines suggested a p value at 0.05 level (*) and solid lines suggested p values at 0.01 level (**). The shaded regions depict the 2 standard errors of $\Delta_{\text{CloudCover}}$ proportion influenced by the surface heating. The numbers in brackets are the minimum and maximum estimated effects associated with the denoted symbols of the statistically significant level. The vertical lines are shown for the distribution of surface heating (in red and purple). Even though the statistical model also includes the negative ΔLST values, the estimated trends are not shown in this case. Only cities with significant ΔCloudCover at p=0.05 level are analyzed.

a. Daytime

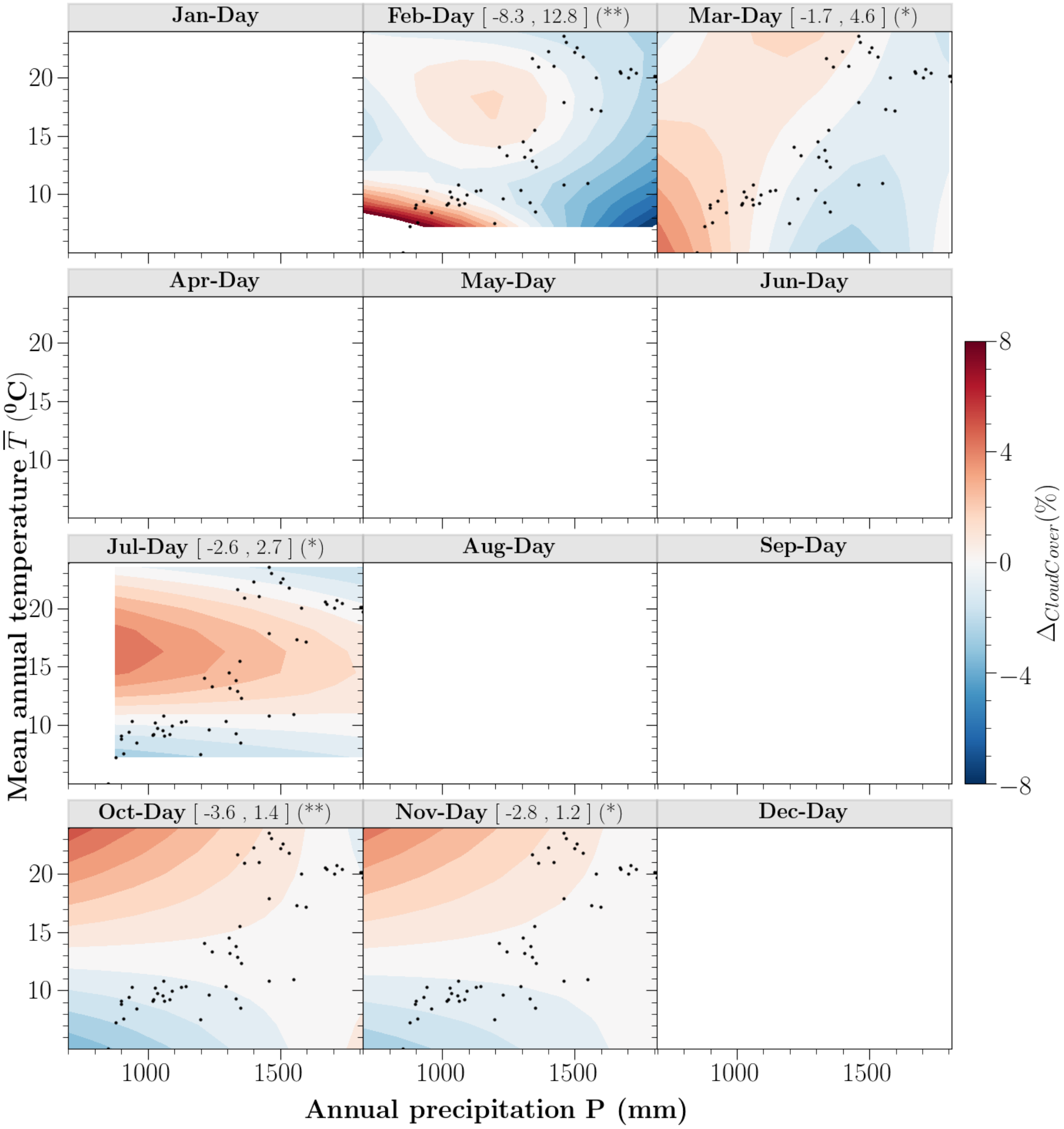

Jan-Day
Feb-Day [-8.3 , 12.8] (**)
Mar-Day [-1.7 , 4.6] (*)
Apr-Day
May-Day
Jun-Day
Jul-Day [-2.6 , 2.7] (*)
Aug-Day
Sep-Day
Oct-Day [-3.6 , 1.4] (**)
Nov-Day [-2.8 , 1.2] (*)
Dec-Day
Mean annual temperature $\overline{T}$ (⁰C)
Annual precipitation P (mm)
$\Delta_{CloudCover}(\%)$
20
15
10
1000
1500
8
4
0
−4
−8

b. Nighttime

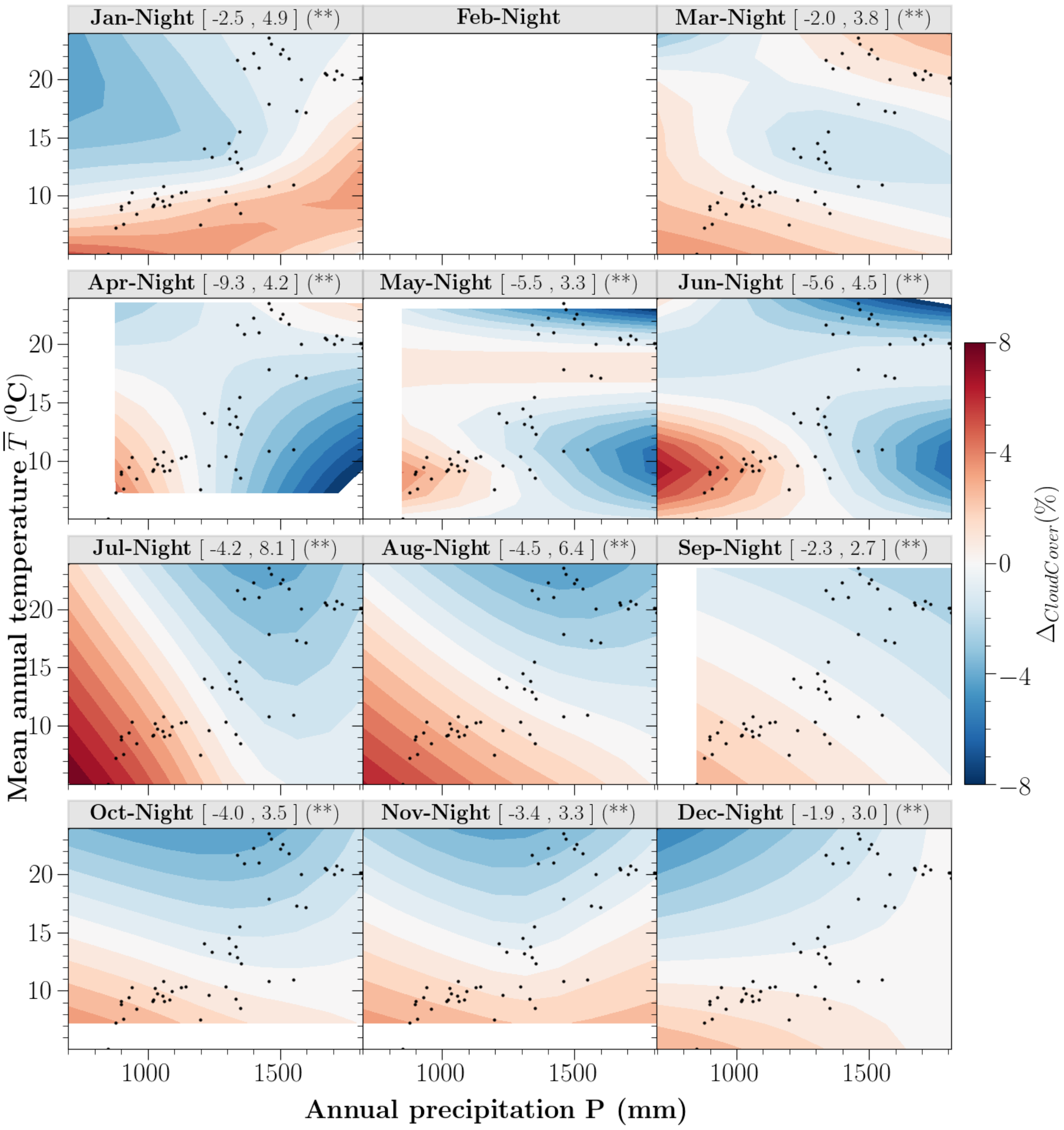

Figure S20. The combined effect (main + interactive effects) of climate background (annual precipitation P, in mm and mean annual temperature $\overline{\boldsymbol{T}}$, in ºC on local cloud patterns ($\Delta_{\text{CloudCover}}$) in GAMs for coastal cities from January to December, for daytime (a) and nighttime (b). Only statistically significant results are illustrated suggested a p value at 0.05 level (*) and at 0.01 level (**). The numbers in brackets are the minimum and maximum estimated effects associated with the denoted symbols of the statistically significant level. Only cities with significant ΔCloudCover at p = 0.05 level are analyzed.

Mountain model. Similar to mesoscale flow assumption as for sea-land breeze, orography can exert persistent and strong circulations (Thunis1996, Sandu2019) that interact with urban areas to further modify the local cloud patterns. Cities located at complex terrains are likely subject to mountain-valley circulations. Thus, we identified these mountainous cities with a greater than 1000 m elevation difference with the highest point within 70 km buffered surrounding areas.

The separate and joint effects of city size (**Figure S22**), surface heating (**Figure S23**) and annual precipitation and mean air temperature (**Figure S24**) are quantified. The coefficient of determination (R^2) values for each month of the mountain model are summarized as follows:

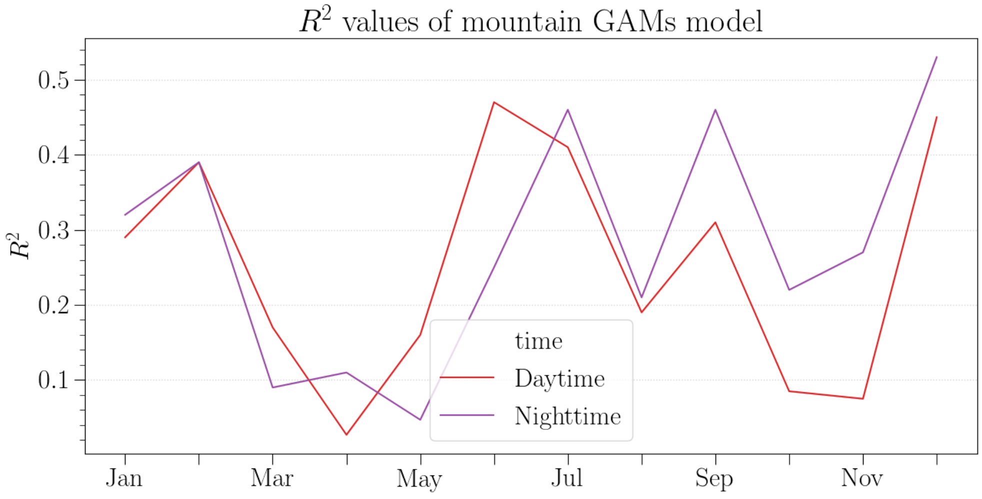

Figure S21. R^2 of models for mountainous cities

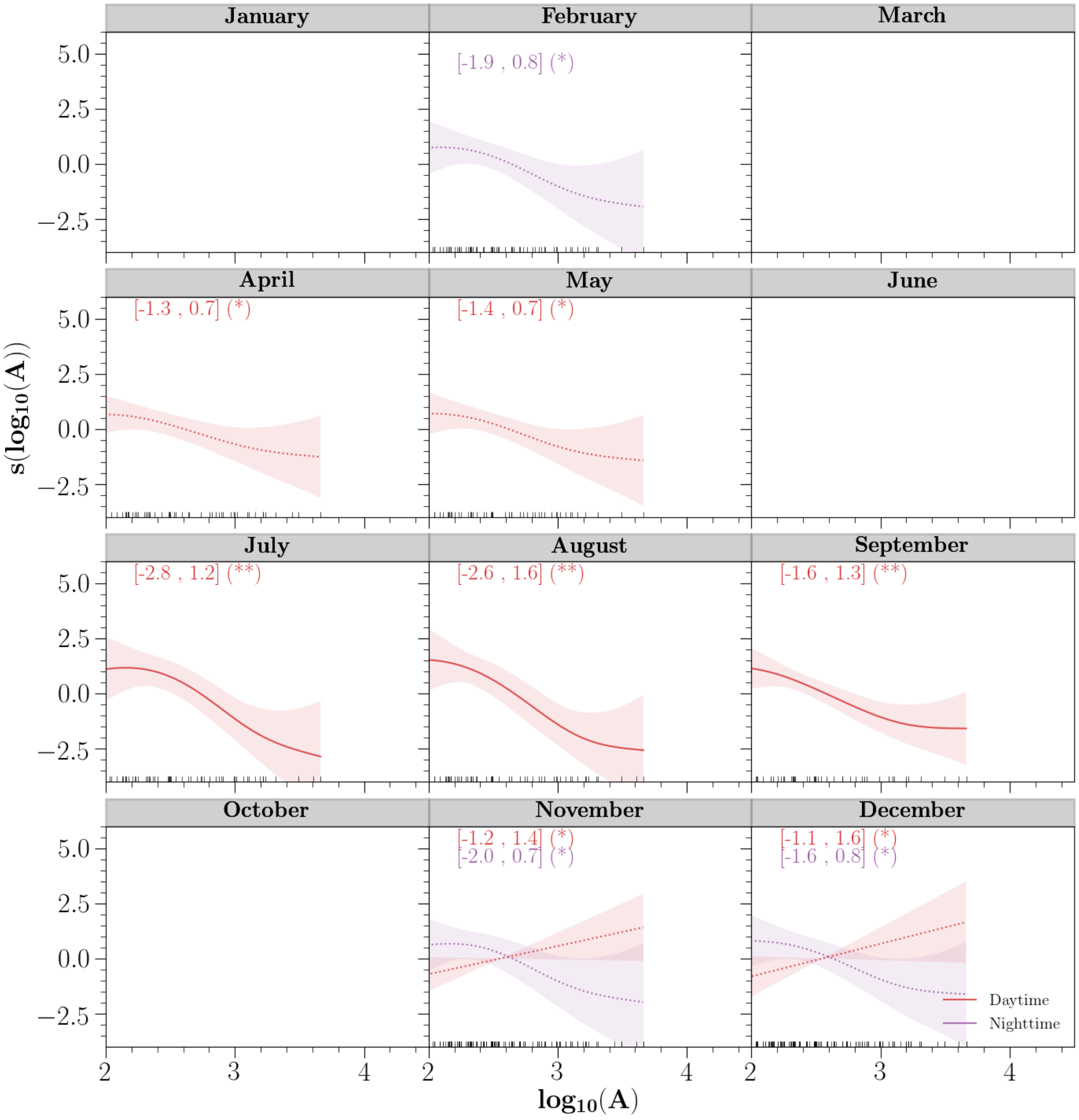

Figure S22. The main effect of city size in log scale ($log_{10}A$, in $log_{10}(km^2)$) on local cloud patterns ($\Delta_{CloudCover}$) in GAMs for mountainous cities from January to December. Only statistically significant results are illustrated and dashed lines suggested a p value at 0.05 level (*) and solid lines suggested p values at 0.01 level (**). The shaded regions depict the 2 standard errors of $\Delta_{CloudCover}$ proportion influenced by the logarithm of city size. The numbers in brackets are the minimum and maximum estimated effects associated with the denoted symbols of the statistically significant level. The vertical black lines are shown for the distribution of city size. The largest mountainous city, e.g., Phoenix Mesa, Arizona ($log_{10}A$ = 3.6) and the smallest inland city, e.g., Delano, California ($log_{10}A$ = 1.6) are considered. Only cities with significant ΔCloudCover at p = 0.05 level are analyzed.

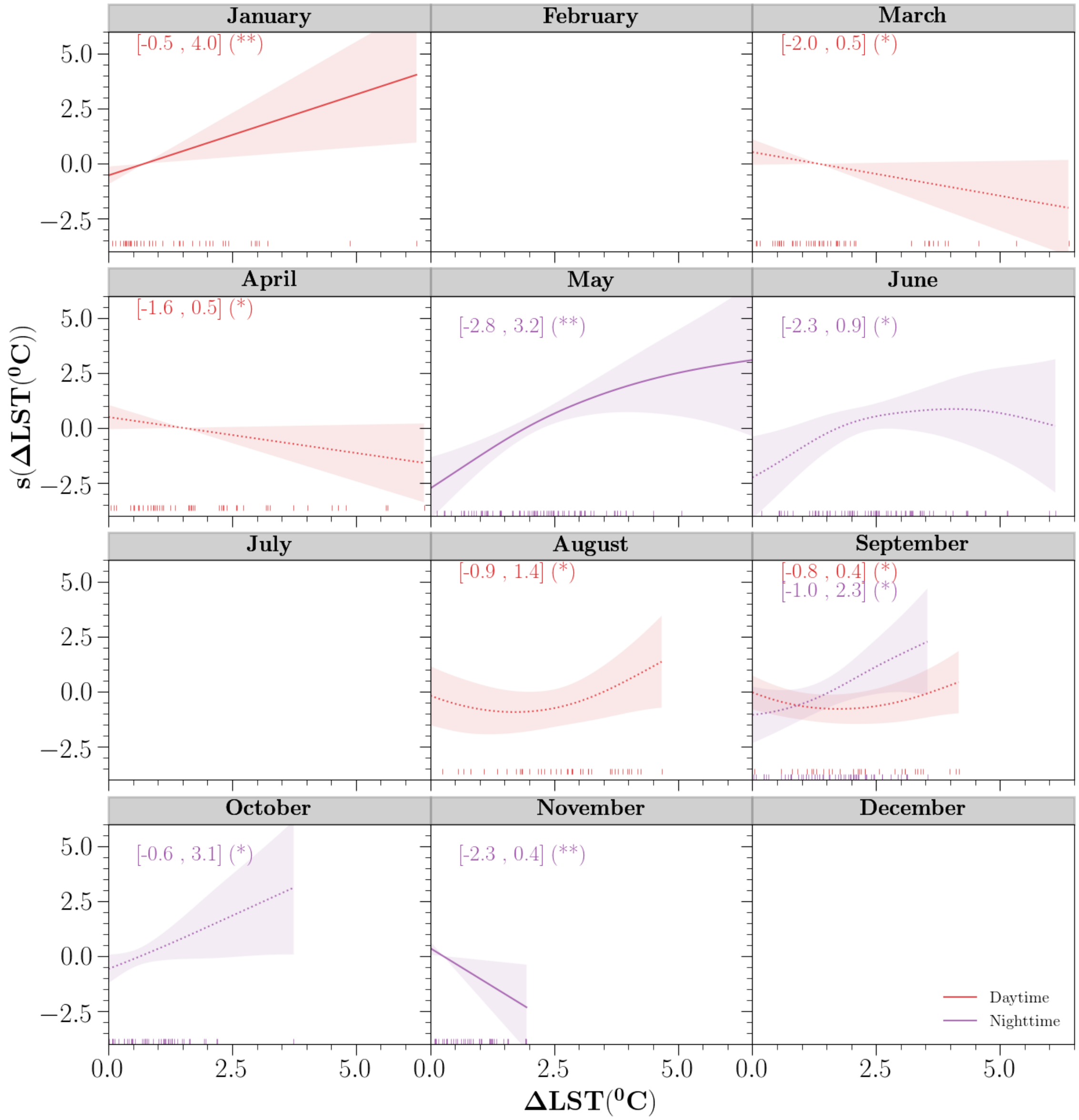

Figure S23. The main effect of surface heating (ΔLST, in ºC) on local cloud patterns ($\Delta_{\text{CloudCover}}$) in GAMs for mountainous cities from January to December. Only statistically significant results are illustrated and dashed lines suggested a p value at 0.05 level (*) and solid lines suggested p values at 0.01 level (**). The shaded regions depict the 2 standard errors of $\Delta_{\text{CloudCover}}$ proportion influenced by the surface heating. The numbers in brackets are the minimum and maximum estimated effects associated with the denoted symbols of the statistically significant level. The vertical lines are shown for the distribution of surface heating (in red and purple). Even though the statistical model also includes the negative ΔLST values, the estimated trends are not shown in this case. Only cities with significant ΔCloudCover at p 0.05 level are analyzed.

a. Daytime

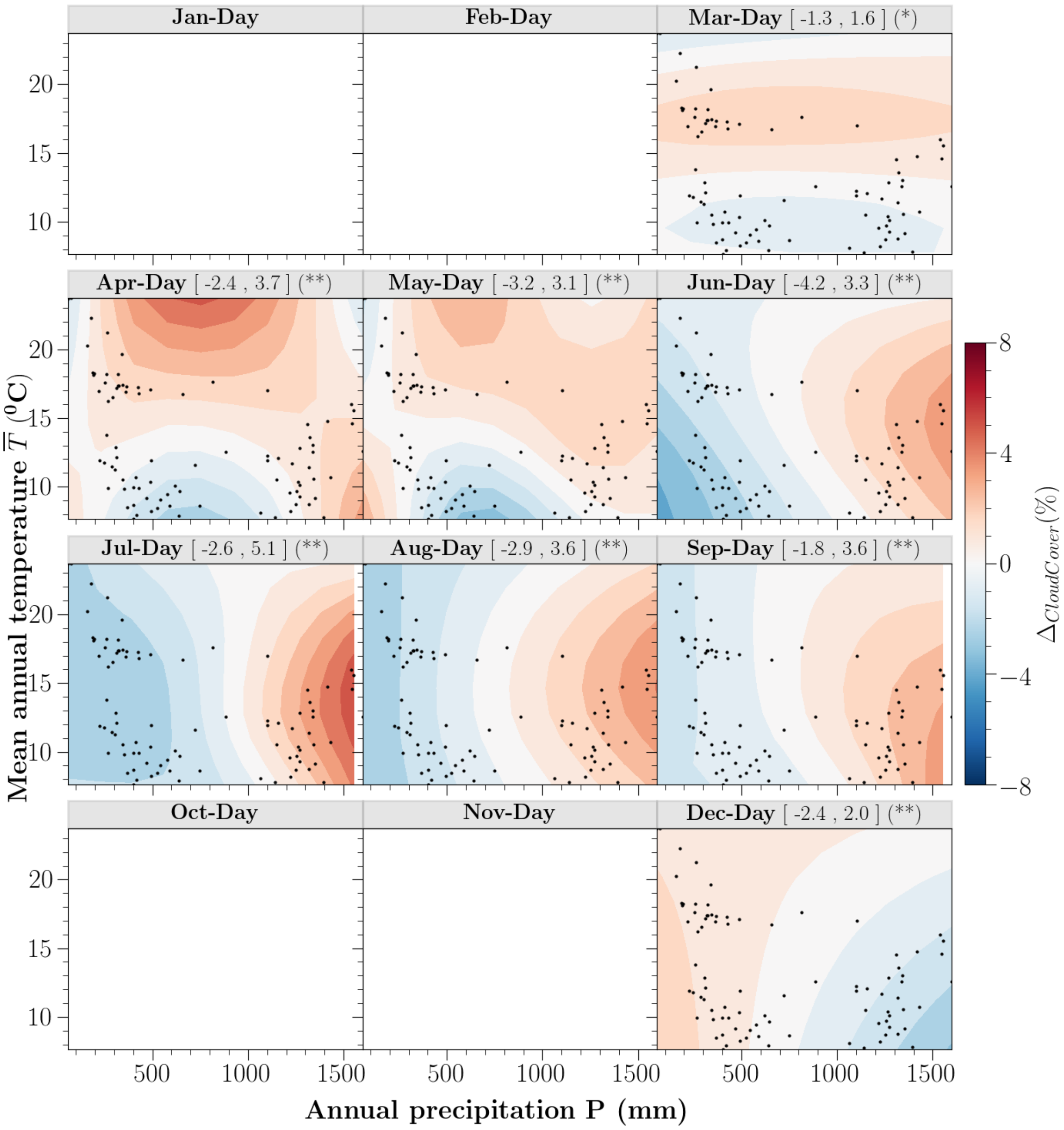

Jan-Day
Feb-Day
Mar-Day [-1.3 , 1.6] (*)
Apr-Day [-2.4 , 3.7] (**)
May-Day [-3.2 , 3.1] (**)
Jun-Day [-4.2 , 3.3] (**)
Jul-Day [-2.6 , 5.1] (**)
Aug-Day [-2.9 , 3.6] (**)
Sep-Day [-1.8 , 3.6] (**)
Oct-Day
Nov-Day
Dec-Day [-2.4 , 2.0] (**)
Mean annual temperature $\overline{T}$ (^{0}C)
Annual precipitation P (mm)
$\Delta_{CloudCover}$(%)

b. Nighttime

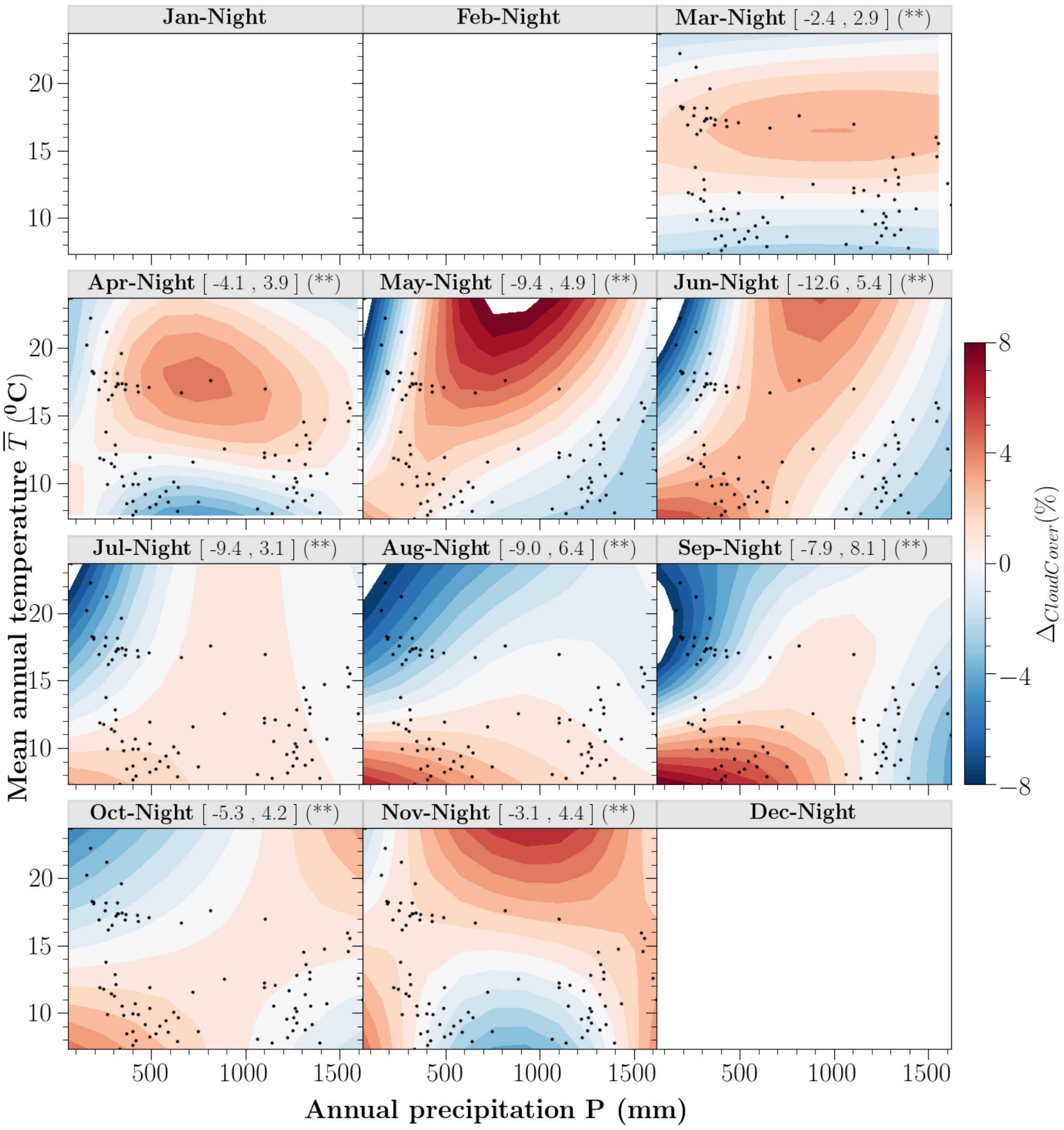

Figure S24. The combined effect (main + interactive effects) of climate background (annual precipitation P, in mm and mean annual temperature $\boldsymbol{\overline{T}}$, in ºC on local cloud patterns ($\Delta_{\text{CloudCover}}$) in GAMs for mountainous cities from January to December, for daytime (a) and nighttime (b). Only statistically significant results are illustrated suggested a p value at 0.05 level (*) and at 0.01 level (**). The numbers in brackets are the minimum and maximum estimated effects associated with the denoted symbols of the statistically significant level. Only cities with significant ΔCloudCover at p = 0.05 level are analyzed.

Section S4

Weekly cycles of urban effects on cloud patterns and the relationship with surface heating

To compare the weekly signal of ΔCloudCover, we grouped and averaged the daily ΔCloudCover signals into two groups: weekdays (Monday - Friday) and weekends (Saturday & Sunday). The t-test results were summarized to reflect the weekdays-weekends contrast in **Figure S25** a,b. The ratios of cities showing significant results are shown in **Figure S26** a,b.

The weekly cycles of urban cloud signals are observed over around 3.2 % of studied domains (**Figure S26** a,b), and the magnitudes and prevalence are considerably weak compared to the diurnal and seasonal signals at the national scale. The weekly cloud cover differences range from -0.23 ~ 0.39 % (median). Its largest signal is found in March nighttime for 0.39 [1.91] % (median [IQR]). The weekly signals are much smaller as compared to diurnal difference (4.42 [1.29] %, daytime and nighttime June) and seasonal difference (7.11 [1.88] %, nighttime June and January). Meanwhile, the weekly variations of ΔLST are small (-0.04 – 0.08 K, median). In the diurnal and seasonal analysis, surface heating often plays less important role than the regional climate, but still shows strong influence particularly during the summer nights (**Fig. 5 ab**). Waste heat emitted from frequent traffics or industrial activities during weekdays can raise temperature more than that during the weekends, leading to weekly cycles of UHI (Jin et al., 2005, Earl et al., 2016). However, little evidence shows there are clear linkages between the weekly cycles of ΔLST and urban cloud signal at the national scale (**Figure S25** a,b). Possible influences from weekly aerosol patterns (Jin et al., 2005, Almeida et al., 2006, Wu et al., 2021) might contribute to the observed weak weekly-cycle of cloud cover, which are not explicitly analyzed in this study.

There are a few cities (**Figure S26** a) with clear weekly cloud cover pattern (about 1.97 [1.47] %) and their corresponding weekday/weekend contrasts in surface heating are about -0.04 [0.58] K (all seasons) (**Figure S26** a,b). For example at nighttime in March, these cities with significant weekly signal reach up to 3.21 [4.92] % differences in cloud cover, the UHI differences are relatively weak by up to 0.15 [0.71] K. The weekly signals of surface heating and the weekly cloud patterns are not consistent across months. For example, despite strong cloud variations during nighttime in other spring and summer month (2.2% to 3.1%, in **Figure S26** b), the weekly variation in surface heating remains negative (-0.08 to -0.3 K, weekend signal is weaker than that in weekdays). In summary, there is no strong evidence to support the potential contribution of anthropogenic activities induced surface heating to the weekly cloud patterns.

In summary, the weekly cycles exhibit in some cities which may be due to the anthropogenic activates, but at the national level, this pattern is not as clear as the diurnal and seasonal contrasts driven by regional climate background as well as other stationary urban surface features.

(a)

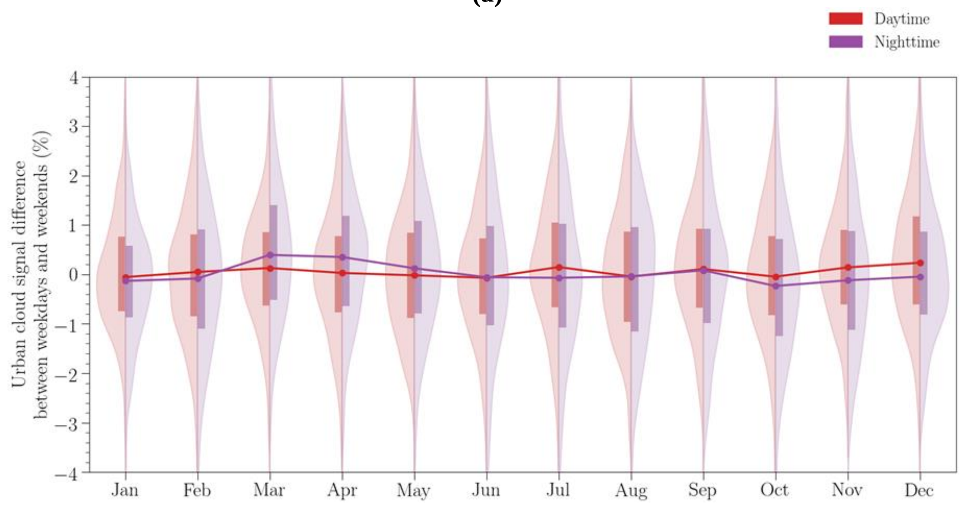

(b)

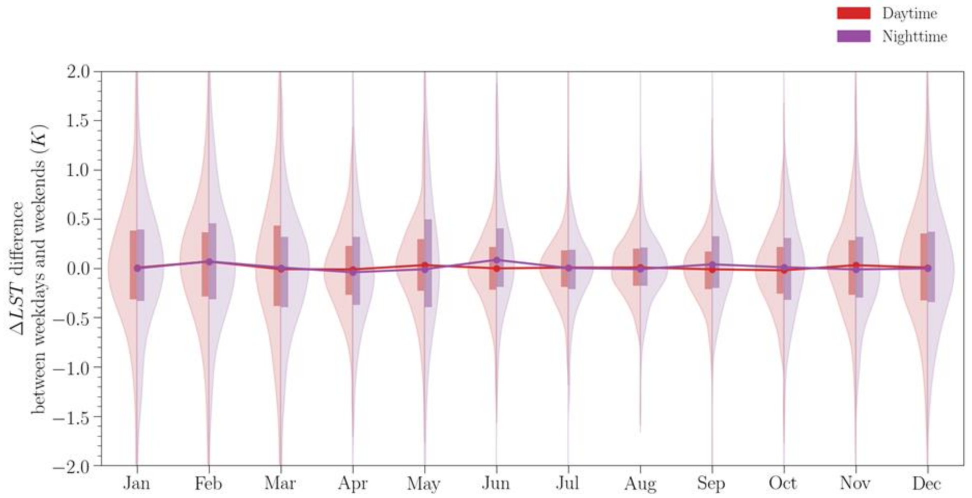

Figure S25. The density curves and boxplots represent the distributions of mean differences in weekdays and weekends (a) ΔCloudCover (%) and (b) ΔLST (K) for day (left) and night (right) of all studied urban domains. The solid lines connect the median values of signals.

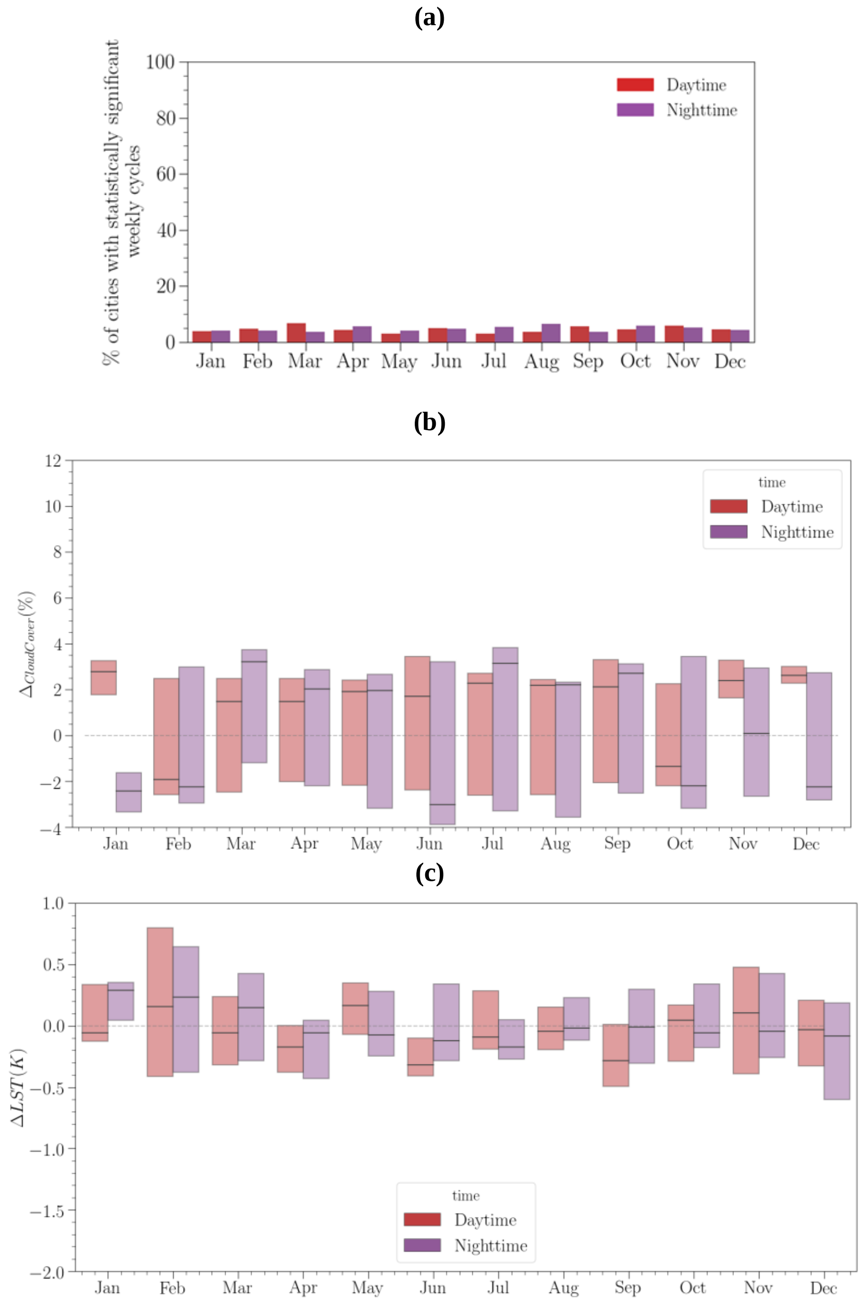

Figure S26. (a) Summary of percentage of the total studied domains showing significant weekly ΔCloudCover contrast between weekdays and weekends; (b) The comparisons of these cities with statistical significant weekly cloud signals and (c) their corresponding weekly signals of surface heating contrast.

Section S5

Definition of urban-background boundaries.

Urban area is defined as the Census Bureau's urban definition which represents densely populated territory. Only urban areas with larger than 50-thousand populations are considered (481 urban areas in total). After merging the contiguous cities, we defined together 447 urban domains. We use the static boundary that is close to the midpoint of 2002-2020 and the urbanization rate is on average about 1.1 % within the 2010 urban boundaries during the entire studied period (see **Figure S29, Section S6** for the urbanization analysis**)**.

The reference domain is selected based on a series of quantitative criteria that apply to all city domains: 1) the reference background is close to the targeted urban domain; 2) the reference domain is physically large enough to represent the regional climate status of cloud patterns; 3) the selected domain is proportional to the urban size. The detailed description of urban-background delineation for an ideal case is shown in **Figure S27**. Generally speaking, the bigger the urban domain, the proportionally larger reference domain is considered. The reference domains are non-urban and non-urban-influenced rural areas (transition zones surrounding urban domain are excluded as well) with no water surfaces (see example, **Figure S28**). The transition and reference background areas are approximately three and five times of the urban area in the ideal case without significant impact from adjacent cities and water surfaces. For cities located in highly urbanized and coastal areas, the exclusion of overlapping urban-background zones from the rural reference of each urban domain can decrease the size of the reference domain. Particularly for coastal highly dense area, the reference domain can be further shrunk by excluding the adjacent water or ocean surfaces. But overall, we verified that (see **Section S7**, **Figure S30**, Tab. S1 for more detail information) for these cases, the rural area after the exclusion still have considerably large spatial extent to represent the cloud patterns.

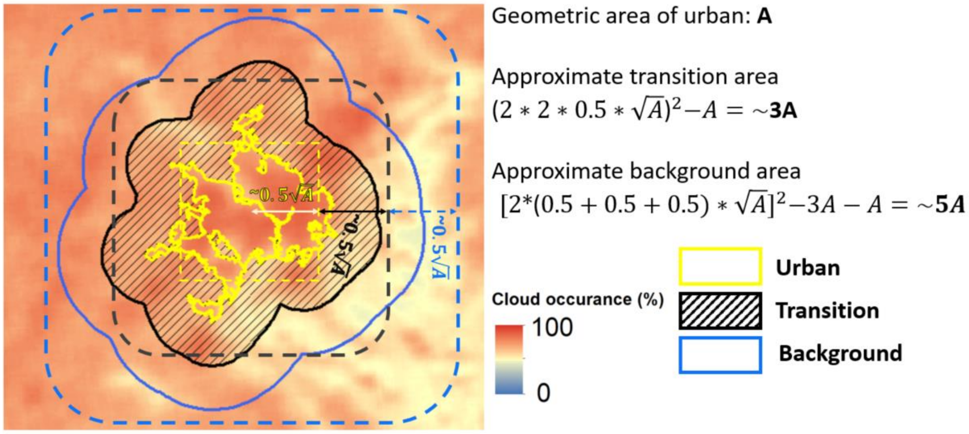

Figure S27. An example of urban-background-transition delineation in the city. Washington, DC--VA--MD is taken as an example. Composite cloud occurrence in nighttime July is overlaid to demonstrate the homogenous cloud patterns over urban and background domains.

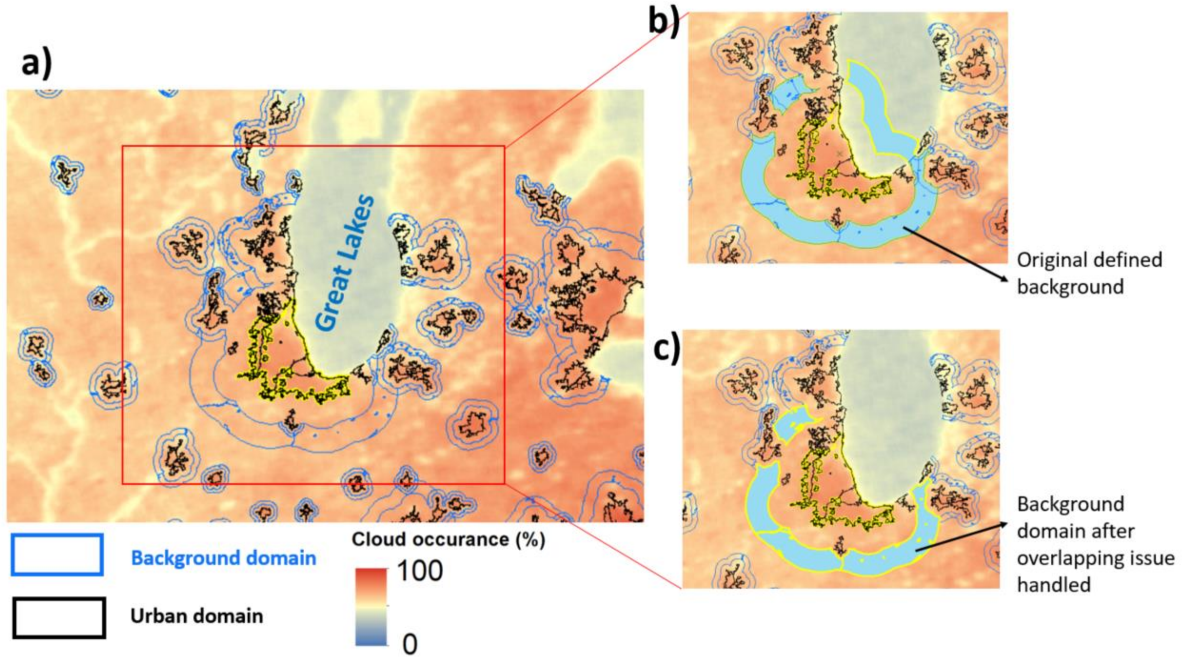

Figure S28. (a) Composite cloud occurrences in daytime July (%) over urban and background domains, taken a highly urbanized area as an example (e.g., Great Lakes region) (b) an example of original defined background and (c) after overlapping urban-background handled for Chicago, IL (highlighted regions).

Section S6

Impact of domain selections (urban domains)

2010 static domain is considered and 18-year mean status is considered for all cities. There may be potential impact of urbanization on cloud signals. Here, MODIS Land Cover Type/Dynamics product MCD12Q1 (Friedl et. al, 2019) (yearly, 500 m spatial resolution) is used to estimate the urbanization rate (hereafter, the percent change of urbanized areas within a time period i.e., 2003 to 2010) for each single city within in census defined domain. We used products in 2003, 2010, and 2020 for this estimation. The early decade suggests CONUS cities have 0.55 +/- 1.08 (mean +/- std) percentage of urbanization within the defined domain (**Figure S29** a). The second decade shows 0.55 +/- 0.91 percentage change in urban expansion relative to the same domain (**Figure S29** b). Over 18 years, the magnitudes of percentage changes in urbanized areas across CONUS cities are overall very small (1.1% on average).

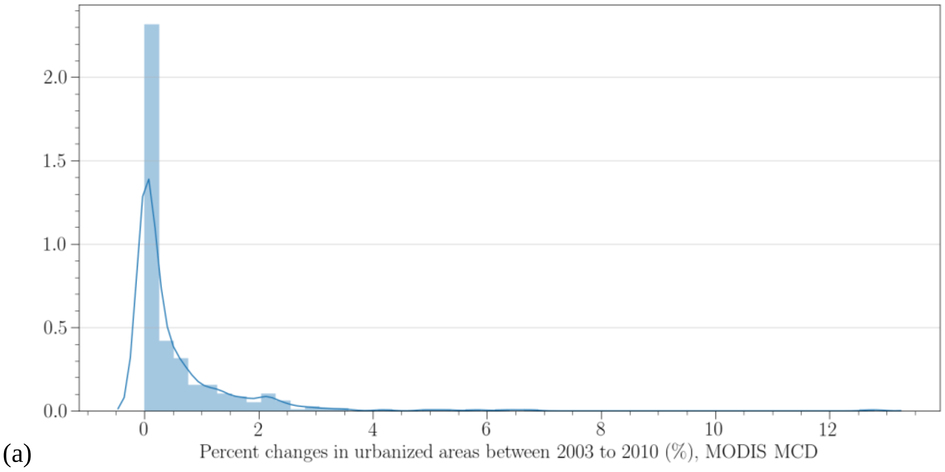

(a)

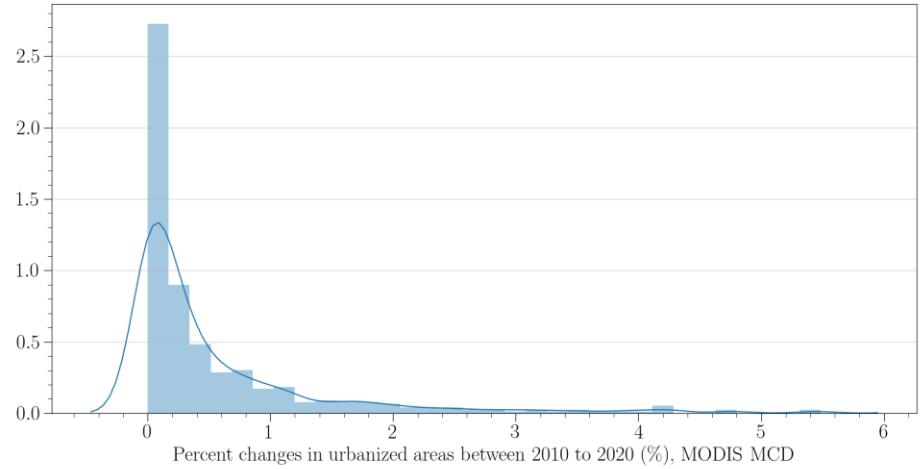

(b)

Figure S29. The distribution of percent changes in urbanized areas between (a) 2003 and 2010 and (b) 2010 and 2020 relative to the 2010 urban area boundary.

Section S7
Impact of domain selections (reference domains)

We excluded the overlapping urban-background zones from the rural references of each urban domain, which can decrease the size of the reference domain. For the scenario with large clustered metropolitan areas, the exclusion of adjacent urban domains can considerably impact the size of reference domain. Particularly for coastal highly dense area, the reference domain can be further shrunk by excluding the adjacent water or ocean surfaces. We designed to have a relatively large reference domain (up to five times of the urban areas) (Figure S27, Section S6), so that allows us to have representative rural signals for most potential scenarios that require exclusion of unrepresentative areas. Table S1 summarized the number of cities have rural reference areas that are equal or less than their corresponding urban domains, as well as their actual coverages of reference domains. First, there are only a small number of cities (around 6% of studied cities, 28 cities) have reference domain smaller than the urban domains. For these cases, their actual spatial extents are still considerably large to represent the cloud pattern. Such as, for Philadelphia, PA--NJ--DE—MD located within highly clustered metropolitan areas, the rural area after the exclusion equals to only half of the urban size, yet, the actual coverage is around 4967 km^2, which is far in the right-heavy-tailed of the distribution of rural areas across all studied cities (**Figure S30**). Also, for large coastal cities in that case, we are cautious to not further expand the reference inland as the sea influence is further weakened, which can decrease the representativeness of the references.

Table S1. Number of cities with rural references area (A_{ref}) lesser or equal to the urban size (A_{urban}). The actual rural coverage (in km^2) range for each group are also labeled. Some examples of cities are labeled with their A_{ref}/A_{urban} and A_{ref} (km^2) within each group.

**Reference size A_{ref}.**	**Number of domains**	**Range of A_{ref} (km^2)**	**Examples of cities (A_{ref}/A_{urban}; A_{ref}, km^2) – sorted by (A_{ref}/A_{urban})**
A_{ref} <= A_{urban}	28 (*)	104.7 – 14229.1	• Philadelphia, PA--NJ--DE—MD (**0.51**; 4967 km^2)<br>• Boston, MA--NH—RI (**0.65**; 10779.5)<br>• Miami, FL (**0.67**; 2620 km^2)<br>• New York--Newark, NY--NJ—CT (**0.77**; 14229 km^2)

(*) Names of cities: Akron, OH, Allentown, PA--NJ, Alton, IL--MO, Bonita Springs, FL, Boston, MA--NH--RI, Bremerton, WA, Concord, CA, Conroe--The Woodlands, TX, Detroit, MI, High Point, NC, Holland, MI, Lorain--Elyria, OH, Los Angeles--Long Beach--Anaheim, CA, Miami, FL, Michigan City--La Porte, IN--MI, Murrieta--Temecula--Menifee, CA, New York--Newark, NY--NJ--CT, Norwich--New London, CT--RI, Philadelphia, PA--NJ--DE--MD, Santa Clarita, CA, Sarasota--Bradenton, FL, Seaside--Monterey, CA, Sebastian--Vero Beach South--Florida Ridge, FL, Spring Hill, FL, Tampa--St. Petersburg, FL, Vineland, NJ, Waldorf, MD, York, PA

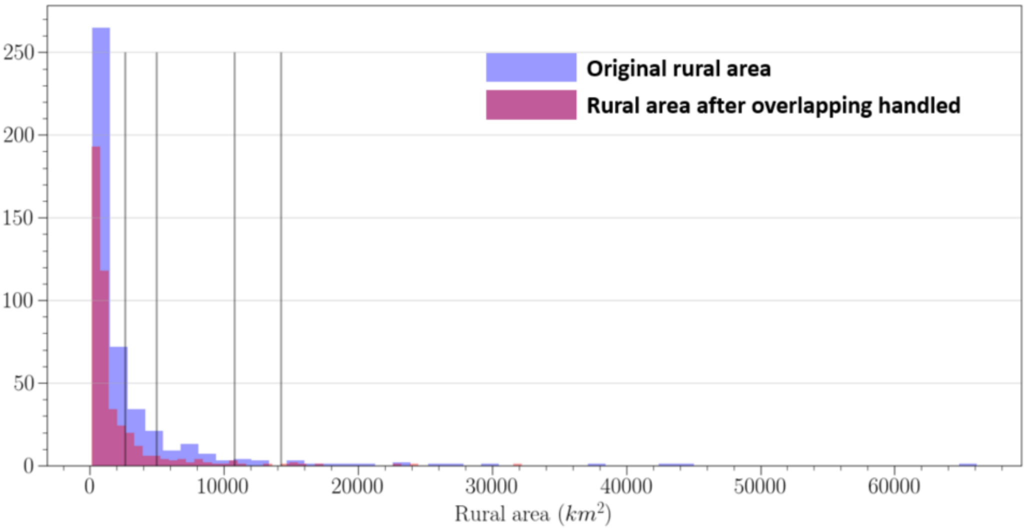

Figure S30. Distribution of rural area across 447 U.S. cities. Woodland, CA has the smallest background area of 104 km^2 and Atlanta, GA has the largest background area of 32,184 km^2. Vertical grey lines show position of some cities with the rural area smaller as compared to the urban area (**Table S1**).

In addition, we conducted the sensitivity test to show the potential impact on ΔCloudCover estimation by increasing the buffer distance of reference area by 10 % and 20 % for all studied cities. Specifically, the new buffer distance of the rural area equals to $1.1 * \sqrt{A}$ *or* $1.2 * \sqrt{A}$ (A is geometric urban area). The results are summarized in **Figure S31**. The relative changes in ΔCloudCover are minor around -0.02 [0.34] (median [IQR]) % for 10% increase in buffer distance and -0.03 [0.51] % for 20% increment of buffer distance in July nighttime (the strongest signal of all time) across all studied cities. Such as, the median value of ΔCloudCover in July nighttime is 5.61% across all studied cities (Fig. 2, main manuscript). For each increasing scenario mentioned, the median of ΔCloudCover can be 5.59 % (for 10% increase) and 5.58 % (for 20% increase), which are very similar to the original value. On average, -0.01 [0.38] for 10% and -0.02 [0.49] % for 20% increase scenario across all months and times (**Figure S32**).

In conclusion, at national level, the impact of increasing the rural area are minor and does not impact the overall estimation of ΔCloudCover. There are a few cities are more sensitive to the reference changes, such as for coastal cities within highly clustered metropolitan areas (**Table S1**). The impact can be up to 0.62 % of change (for 10 % increase) and 1.38 % (for 20% increase) for Philadelphia, PA--NJ--DE—MD as compared to the national level (**Figure S31**). But there only a few cities in that case, and also note the influence on an expansion of reference areas further inland on the urban signals as well.

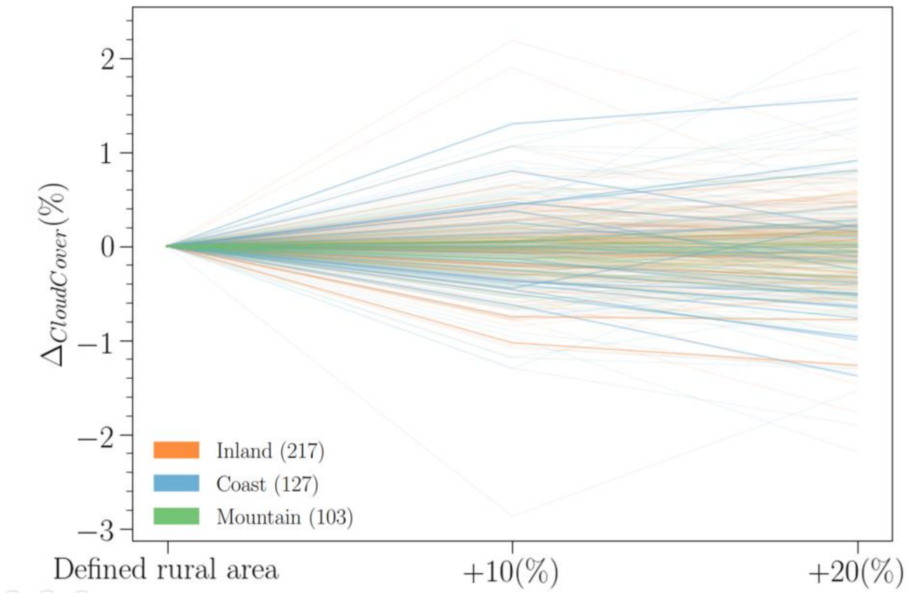

Figure S31. Relative change in ΔCloudCover by increase the buffer distance by 10 and 20 % to the currently used buffer distance for the reference domain. Each line represents for the ΔCloudCover for each city for July nighttime. Cities with rural areas smaller than the urban size (Table S1) are highlighted as darker-color lines.

(a)

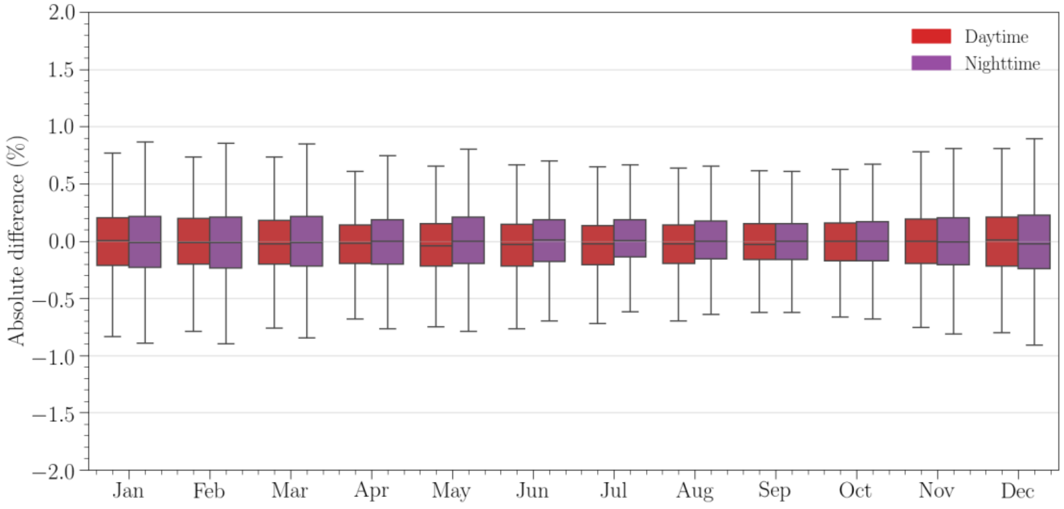

(b)

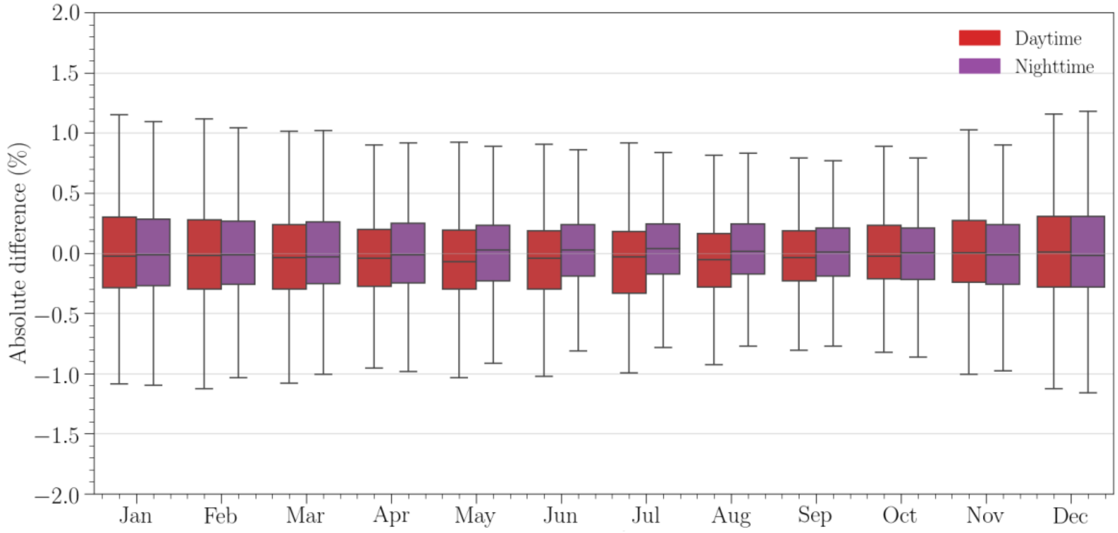

Figure S32. Distribution of change in ΔCloudCover when increase a) 10% and b) 20 % buffer distance of the reference area across all studied cities.

Section S8
Characterization of sequence of Terra clear-sky LST (used as indicator for surface heating) and Aqua cloudy images

LST is a direct measure of outgoing long-wave radiation, and this measurement straightforwardly reflects differential surface heating in each domain. Due to the uneven cloud distribution over the urban-background domain, we only consider the clear-sky LST images across each domain (i.e., images with at least 98% clear-sky pixels covered over each domain). The 18-year clear-sky temporal composite LST is estimated in a given month and then we calculated the urban-background differences using the same spatial criteria for cloud cover estimation. We acknowledged the limitation of satellite LST that are only under clear skies. Thus, we try to adopt an earlier overpass of observations which can potentially initialize the favorable conditions for cloud formation. We therefore characterize how often the MODIS Terra LST as followed by MODIS Aqua cloud cover from 18-year historical observations in this section. Typically, if most of the time, an Terra clear-sky LST followed by an Aqua cloudy images, the surface heating from Terra LST is reasonable to justify the contribution of surface heating to the possible cloud formation.

Specifically, we counted the occurrences (in frequency) within a month over 18 years in each given city with the scenario with the clear-sky Terra LST followed with cloudy condition in Aqua overpass. The national summary of all cities is shown in **Figure S33**. There are higher chances (93% on average)to have clear morning (evening) followed by the cloudy afternoon (mid-night) scenarios from all studied cases (compared to chances with both clear-sky conditions).

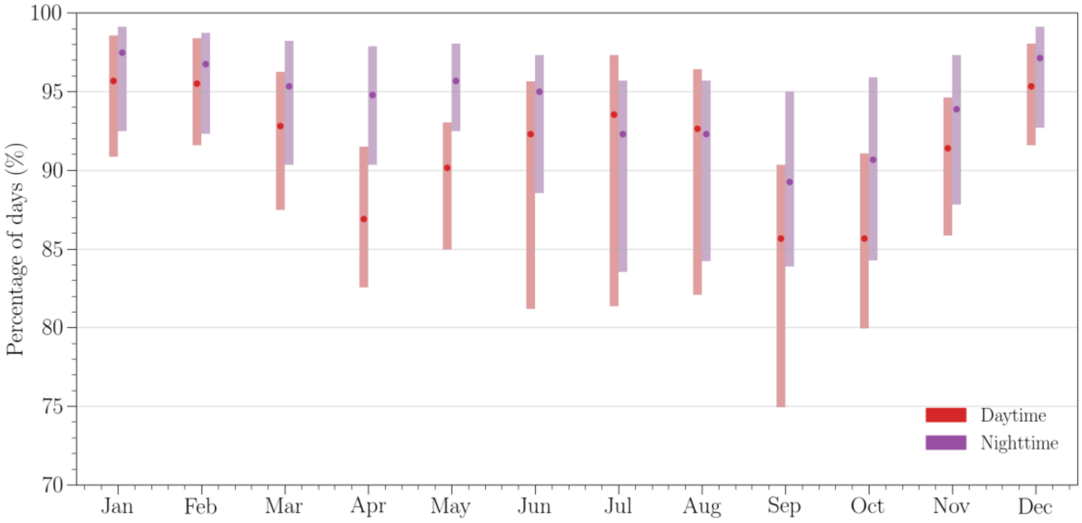

Figure S33. Distribution of how often clear-sky Terra LST followed by cloudy Aqua images across all studied cities. Dots show the median of percentages of days and the boxes show the IQR ranges.

SI References

1. Friedl, M., D. Sulla-Menashe. *MCD12Q1 MODIS/Terra+Aqua Land Cover Type Yearly L3 Global 500m SIN Grid V006*. 2019, distributed by NASA EOSDIS Land Processes DAAC, https://doi.org/10.5067/MODIS/MCD12Q1.006. Accessed 2022-12-23.
2. N. Earl, I. Simmonds, and N. Tapper, "Weekly cycles in peak time temperatures and urban heat island intensity," Environ. Res. Lett., vol. 11, no. 7, 2016.
3. T. Wu and B. E. Boor, "Urban aerosol size distributions: A global perspective," Atmos. Chem. Phys., vol. 21, no. 11, pp. 8883–8914, 2021.
4. S. M. Almeida, C. A. Pio, M. C. Freitas, M. A. Reis, and M. A. Trancoso, "Source apportionment of atmospheric urban aerosol based on weekdays/weekend variability: Evaluation of road re-suspended dust contribution," Atmos. Environ., vol. 40, no. 11, pp. 2058–2067, 2006.
5. T. R. Oke, G. Mills, A. Christen, and J. A. Voogt, Urban climates. Cambridge University Press, 2017.
